# Genomic and functional characterization of five novel *Salmonella*-targeting bacteriophages

**DOI:** 10.1186/s12985-021-01655-4

**Published:** 2021-09-08

**Authors:** Marta Kuźmińska-Bajor, Paulina Śliwka, Maciej Ugorski, Paweł Korzeniowski, Aneta Skaradzińska, Maciej Kuczkowski, Magdalena Narajaczyk, Alina Wieliczko, Rafał Kolenda

**Affiliations:** 1 Department of Biotechnology and Food Microbiology, Faculty of Biotechnology and Food Sciences, Wrocław University of Environmental and Life Sciences, Wrocław, Poland; 2 Department of Biochemistry and Molecular Biology, Faculty of Veterinary Medicine, Wrocław University of Environmental and Life Sciences, Wrocław, Poland; 3 Department of Epizootiology and Clinic of Birds and Exotic Animals, Faculty of Veterinary Medicine, Wrocław University of Environmental and Life Sciences, Wrocław, Poland; 4 Department of Electron Microscopy, Faculty of Biology, University of Gdansk, Gdansk, Poland

**Keywords:** *Salmonella enterica*, Bacteriophages, Bacteriophage genetics, Comparative genomics

## Abstract

**Background:**

The host-unrestricted, non-typhoidal *Salmonella enterica* serovar Enteritidis (*S.* Enteritidis) and the serovar Typhimurium (*S.* Typhimurium) are major causative agents of food-borne gastroenteritis, and the host-restricted *Salmonella enterica* serovar Gallinarum (*S.* Gallinarum) is responsible for fowl typhoid. Increasing drug resistance in *Salmonella* contributes to the reduction of effective therapeutic and/or preventive options. Bacteriophages appear to be promising antibacterial tools, able to combat infectious diseases caused by a wide range of *Salmonella* strains belonging to both host-unrestricted and host-restricted *Salmonella* serovars.

**Methods:**

In this study, five novel lytic *Salmonella* phages, named UPWr_S1-5, were isolated and characterized, including host range determination by plaque formation, morphology visualization with transmission electron microscopy, and establishment of physiological parameters. Moreover, phage genomes were sequenced, annotated and analyzed, and their genomes were compared with reference *Salmonella* phages by use of average nucleotide identity, phylogeny, dot plot, single nucleotide variation and protein function analysis.

**Results:**

It was found that UPWr_S1-5 phages belong to the genus *Jerseyvirus* within the *Siphoviridae* family. All UPWr_S phages were found to efficiently infect various *Salmonella* serovars. Host range determination revealed differences in host infection profiles and exhibited ability to infect *Salmonella enterica* serovars such as Enteritidis, Gallinarum, Senftenberg, Stanley and Chester. The lytic life cycle of UPWr_S phages was confirmed using the mitomycin C test assay. Genomic analysis revealed that genomes of UPWr_S phages are composed of 51 core and 19 accessory genes, with 33 of all predicted genes having assigned functions. UPWr_S genome organization comparison revealed 3 kinds of genomes and mosaic structure. UPWr_S phages showed very high sequence similarity to each other, with more than 95% average nucleotide identity.

**Conclusions:**

Five novel UPWr_S1-5 bacteriophages were isolated and characterized. They exhibit host lysis range within 5 different serovars and are efficient in lysis of both host-unrestricted and host-restricted *Salmonella* serovars. Therefore, because of their ability to infect various *Salmonella* serovars and lytic life cycle, UPWr_S1-5 phages can be considered as useful tools in biological control of salmonellosis.

**Supplementary Information:**

The online version contains supplementary material available at 10.1186/s12985-021-01655-4.

## Background

*Salmonella enterica* is one of the major causative agents of human gastrointestinal infections from contaminated food of animal origin. The severity of salmonellosis in humans varies from mild symptoms to life-threatening conditions, depending on various factors including the properties of the infecting serovars and their host specificity [1–3]. Two host-unrestricted *Salmonella enterica* serovars, Enteritidis and Typhimurium, commonly isolated from poultry products, are the most frequent causes of acute gastroenteritis [4, 5]. Therefore, continuous growth of poultry eggs and meat production and consumption lead to increasing public health threats. On the other hand, the host-restricted *Salmonella enterica* serovar Gallinarum infecting only avian species causes fowl typhoid characterized by high morbidity and mortality, which often leads to a severe septicemic disease, and is responsible for considerable economic losses in the poultry industry worldwide [6, 7].

A major worldwide problem in combatting salmonellosis is the rapidly growing antibiotic resistance [8]. The prevalence of antibiotic-resistant *Salmonella* strains poses a serious threat to public health as a source of untreatable infections and epidemics. Therefore, it is necessary to develop and apply new strategies to prevent and control these infections. One such approach is phage therapy, proposed as an important alternative to antibiotic treatment and as a preventive strategy in human infections and food production [9, 10]. Bacteriophages are self-replicating and self-limiting bacterial viruses since they multiply only at the site where the host is located and are eliminated gradually in the absence of host bacteria [11]. Low inherent toxicity, lack of cross-resistance with antibiotics and versatility are the advantages of phages in both therapy and food safety [12, 13]. However, bacteriophages are host-specific and often infect only one *S. enterica* serovar [14, 15], which in many instances may be a limiting factor in pathogen elimination, as new phages have to be identified for each serovar or even strain causing an epidemic or outbreak [16]. Therefore, bacteriophages used as a preventive strategy or as an antimicrobial tool should show lytic activity against a wide range of pathogens. In the case of *Salmonella*, such broad-host-range bacteriophages were described as those that infected at least several serovars. However, the term broad-host-range *Salmonella* phages was used for phages able to infect 27 *Salmonella* serovars [17] as well as only three *Salmonella* serovars [18]. It was also shown that *Salmonella* phages were able to lyse *Escherichia coli* and *Klebsiella oxytoca* strains [19] as well as strains belonging to the species *Enterobacter cloacae* and *Cronobacter sakazakii* [20].

Considering the great potential of bacteriophages as antimicrobial agents in *Salmonella* eradication, this study was undertaken to isolate and characterize bacteriophages against a wide spectrum of *S. enterica* serovars. As a result, five novel bacteriophages, named UPWr_S1-5, able to infect numerous *Salmonella* strains, including these belonging to the host-unrestricted serovar *S*. Enteritidis and host-restricted *S*. Gallinarum were isolated and characterized.

## Material and methods

### Bacterial strains, phages and growth conditions

*Salmonella enterica* strains (63) used in this study were obtained from the Strain Collection of the Department of Epizootiology and Clinic of Bird and Exotic Animals, Wrocław University of Environmental and Life Sciences. All bacterial strains were cultivated in Luria–Bertani (LB) broth (Sigma-Aldrich, Germany) under aerobic conditions at 37 °C with agitation. The lysogenic phage P22 (DSM 18523), the strictly lytic phage Felix O1 (DSM 18523) and *S.* Typhimurium LT2 (DSM 18522) as their host were obtained from the German Collection of Microorganisms and Cell Cultures GmbH (DSMZ, Braunschweig, Germany). The phages and *S.* Typhimurium LT2 reconstitution and propagation were conducted in accordance with the supplier’s instructions.

### Bacteriophage isolation and purification

One hundred eighty-four samples, including feces, litter and manure from poultry farms, drainage ditches located near poultry farms or treatment plants were collected from 2015 to 2016. For bacteriophage isolation, 5 g of a solid sample or 5 ml of a liquid sample was mixed with 15 ml of LB broth, inoculated with five randomly chosen *Salmonella* strains and incubated overnight at 37 °C with agitation. Bacterial cultures were centrifuged and supernatants containing phages were filtered using a 0.22 µm filter (Merck Millipore, USA). The presence of phages was assessed using a spot test. For this purpose, overnight cultures of *Salmonella* were spread on LB agar plates and incubated for 40 min at room temperature. Serial dilutions of filtered supernatants containing phages were spotted onto the surface of the plates, left to dry and incubated at 37 °C overnight or until a visible bacterial lawn grew. Plates were inspected for lysis zones or the presence of plaques. Clear, single and well-separated plaques were picked and eluted into 200 µl of LB broth culture. Phage suspension was added to 5 ml of the fresh host culture and incubated overnight. To obtain a single phage preparation, each bacteriophage was purified using five consecutive rounds of single-plaque picking and propagation.

### Bacteriophage amplification and titer determination

On the basis of clear plaque formation and efficient propagation, *S*. Enteritidis A41 and A28 were used as hosts for phages UPWr_S1 and UIPWr_S5, respectively, whereas *S*. Enteritidis A36 was employed as a host for UPWr_S2, UPWr_S3 and UPWr_S4. The propagation of isolated bacteriophages was performed on the respective host strain. The phages were propagated according to Oliveira et al. [21] with slight modifications. The bacterial culture was prepared by inoculation of 10 ml of LB broth with a single colony following overnight incubation at 37 °C with agitation. In short, 0.5 ml of overnight culture was inoculated into 10 ml of fresh LB broth and cultivated for 2 h. Then, 5 ml of phage suspension was added and culture was continued overnight at 37 °C. In the next step, the bacterial culture was centrifuged for 10 min at 5000×*g* to remove any remaining cell debris and filtered through 0.22 µm pore size syringe filters. The resulting phage suspension was again added to 100 ml of fresh host culture and incubated overnight at 37 °C. The centrifugation and filtration steps were repeated. Bacteriophage titer was determined using routine test dilution and double-agar overlay [22].

### Host-range determination and efficiency of plating

To evaluate the lytic spectrum of the isolated bacteriophages, a spot test was employed by dropping high titer phage lysates (10^12^–10^14^ plaque forming units (PFU)/ml) onto agar plates with *Salmonella* strains. The plates were incubated at 37 °C for 24 h and examined for the degree of clearing zones.

For efficiency of plating (EOP), the spot test was conducted with phage lysates diluted serially 10 times and spotted on agar plates with phage-sensitive *Salmonella* strains [23]. The EOP was calculated as the ratio of PFU formed by phages infecting *Salmonella* strains to PFU formed on a propagation host. All the experiments were carried out in triplicate. EOP values were defined as high when EOP ≥ 0.5, moderate when 0.01 ≤ EOP < 0.5 and low when 0.0001 < EOP < 0.01 [24]. The data were reordered by hierarchical clustering analysis using the complete linkage method and R software [25]. Next, a heatmap was generated with the package ggplot2 implemented in the R software [26]. The R code is provided in Additional file 1: File S1.

### Morphological classification

To ascertain the morphology of high titer phage samples, 5 µl of phage suspension was adsorbed onto 400 copper mesh grids (Sigma-Aldrich) coated with 2% collodion solution (Sigma-Aldrich) and carbon for 3 min, stained with 2% uranyl acetate (pH 4.5) (BDH Chemicals, UK) for 15 s and air-dried. Electron microscopic analysis was performed at 120 kV using a Tecnai G2 Spirit BioTWIN transmission electron microscope (FEI). Micrographs were taken at 250,000 times magnification, with Olympus Soft Imaging Solution software.

### Mitomycin C assay

In order to determine the phage life cycle, the mitomycin C assay was performed according to Owen et al., [27] with some modifications. Briefly, for prophage induction, the temperate phage P22, the strictly lytic phage Felix O1 and their host *S.* Typhimurium LT2 were used as positive and negative controls, respectively. Phages were spotted on the fresh bacterial lawns of respective hosts and incubated overnight at 37 °C. After incubation, the presence of resistant bacterial clones was observed in lytic zones. At least 10 phage-resistant colonies were picked from one plate and purified by fivefold subculturing on MacConkey agar (Sigma-Aldrich) in order to remove attached phage particles. To confirm phage resistance of these bacterial strains, a standard spot test was performed. For chemical induction of phages from phage-resistant strains, 100 ml of LB broth was inoculated with overnight bacterial cultures and cultivated until an optical density at 600 nm of 0.2 was reached. To stimulate prophage induction, mitomycin C (Sigma-Aldrich) was added to the final concentration of 1 µg/ml. As a negative control each of the analyzed bacterial cultures was grown in the absence of mitomycin C. Overnight cultures, both with and without mitomycin C induction, were centrifuged at 4,000 × g, filtered through 0.22 µm filter and spotted on cultured Petri dishes with the appropriate *Salmonella* host. After overnight incubation at 37 °C, plates were analyzed for the presence of clear zones.

### One-step growth curve, latent period and burst size

The adsorption assay was carried out according to Rahman et al. [28] with minor modifications. The log phase cultures of host strains were infected with phage suspensions at optimal multiplicity of infection (MOI) and incubated at 37 °C. At 0, 3, 6, 9, 12, 15, and 18 min of incubation, aliquots were taken (100 µl), mixed with LB medium (900 µl), and immediately filtered through a 0.22 µm pore size syringe filter. The titer of unadsorbed phage particles was determined by the double-agar layer method. The experiment was repeated three times for each phage.

To determine the latent period and phage burst size, a one-step growth curve was performed according to Yu et al. [29]. Phage suspensions were mixed with exponential growth phase cultures of host bacteria at optimal MOI. Samples were taken at 10 min intervals over a period of 80 min and phage titer was determined via the double-agar layer method. Latent time was measured as the interval between phage adsorption and the liberation of phage particles. Burst size was calculated as the ratio of the phage titer at the plateau phase to the initial count of infected bacterial cells/initial phage titer. Each experiment was performed in triplicate**.**

### Adsorption curve

To determine the adsorption rate, *Salmonella* strains were grown in LB medium to the exponential phase, then infected with UPWr_S1, UPWr_S4 and UPWr_S5 phages at MOI 1 and UPWr_S2 and UPWr_S3 phages at MOI 0.1 and incubated at 37 °C. Samples were taken at 1, 2, 3, 4, 5, 6, 7, 8, 9, 10, 15 and 18 min and centrifuged. The supernatants were used for plaque assays to determine the titers of non-adsorbed phages [30]. This experiment was repeated three times independently.

### Bacteriophage DNA extraction, genome sequencing, assembly and annotation

High-titer phage suspensions (10^12^–10^14^ PFU/ml) were used for DNA extraction. Prior to the extraction, bacterial lysates containing phages were treated with DNase I (80 U/ml; Thermo Scientific, USA) and RNase I (80 μg/ml; Thermo Scientific, USA) at 37 °C for 3 h to remove non-phage nucleic acids. Phage DNA was then extracted using a High Pure Viral Nucleic Acid Large Volume Kit (Roche, Mannheim, Germany) with initial phage capsid disruption by treatment with proteinase K and 0.5% sodium dodecyl sulfate (Sigma-Aldrich) for 2 h at 56 °C. The integrity of the extracted DNA was determined by electrophoresis in 0.7% agarose stained with Midori Green DNA Stain (Nippon Genetics Europe, Germany). The concentration of DNA was determined with a Biowave II UV/Vis spectrophotometer (Biochrom WPA, UK) and purity was determined in terms of the ratio 260/280 nm. Phage genomes were sequenced with the Illumina MiSeq next-generation sequencing platform (Genomed SA. Poland) using MiSeq Reagent Kit v2 500-cycles (Illumina, USA). Sequencing quality was assessed on the basis of average base quality, GC content and adapter contamination [31]. All sequenced phages were assembled into one unique contig and sequence assembly was conducted with the Shovill pipeline and assembly improvement pipeline [32]. Genome assemblies were annotated with Prokka [33].

#### Comparison, clustering and analysis of phage genomes

Phage genomes were characterized by overall genome BLAST similarities to the 95 *Salmonella* phage genomes available at the NCBI. The average nucleotide identity (ANI) of the phage genomes was analyzed using PYANI (v0.2.9) [34]. Genome sequence comparisons were generated with BLASTn and visualized with EasyFig software [35]. The predicted functions of the open reading frames (ORFs) were analyzed for UPWr_S1, UPWr_S2 and UPWr_S5 phages by BLASTn [36] and BLASTp [37] searches, with a cut-off *E* value of 10^–4^. Putative protein sequences were analyzed by BLAST/HMMer Pfam [38] description and Phyre 2.0/HHpred prediction [39, 40] for conservative domain identification. The search of putative tRNA-encoding genes was conducted using ARAGORN [41] as part of the Prokka annotation process [42]. For evolutionary relationships, 60 available genomes of bacteriophages belonging to *Jerseyvirus* and six available genomes of *Cornellvirus* bacteriophages, both members of the *Guernseyvirinae* subfamily, *Siphoviridae* family, and 29 representative genomes from other phage genera infecting *Salmonella* were selected from the GenBank Virus database. In contrast to phages from *Jerseyvirus* and *Cornellvirus* genera within *Guernseyvirinae* subfamily, phages belonging to genus *Kagunavirus* due to their specificity to *Escherichia coli* were not included in the analysis. All phages used in the analysis are listed in Additional file 2: Table S1. All pairwise comparisons of the nucleotide sequences were conducted using the Genome-BLAST Distance Phylogeny (GBDP) method under settings recommended for prokaryotic viruses, using VICTOR software. The resulting intergenomic distances were used to infer a balanced minimum evolution tree with branch support via FASTME including subtree pruning and regrafting (SPR) postprocessing. Branch support was inferred from 100 pseudo-bootstrap replicates each. Information about genus and family for each sequence was added using iTOL [43, 44]. The whole genomes were also compared using a dot plot analysis implemented in FlexiDot [45].

#### Sequence accession numbers

The annotated UPWr_S phage genome sequences were deposited in GenBank under accession numbers MT588083, MT632017, MT632018, MT632019 and MT632020 for UPWr_S1, UPWr_S2, UPWr_S3, UPWr_S4 and UPWr_S5, respectively.

## Results

### Bacteriophage host range and EOP determination

**Fig. 1:**
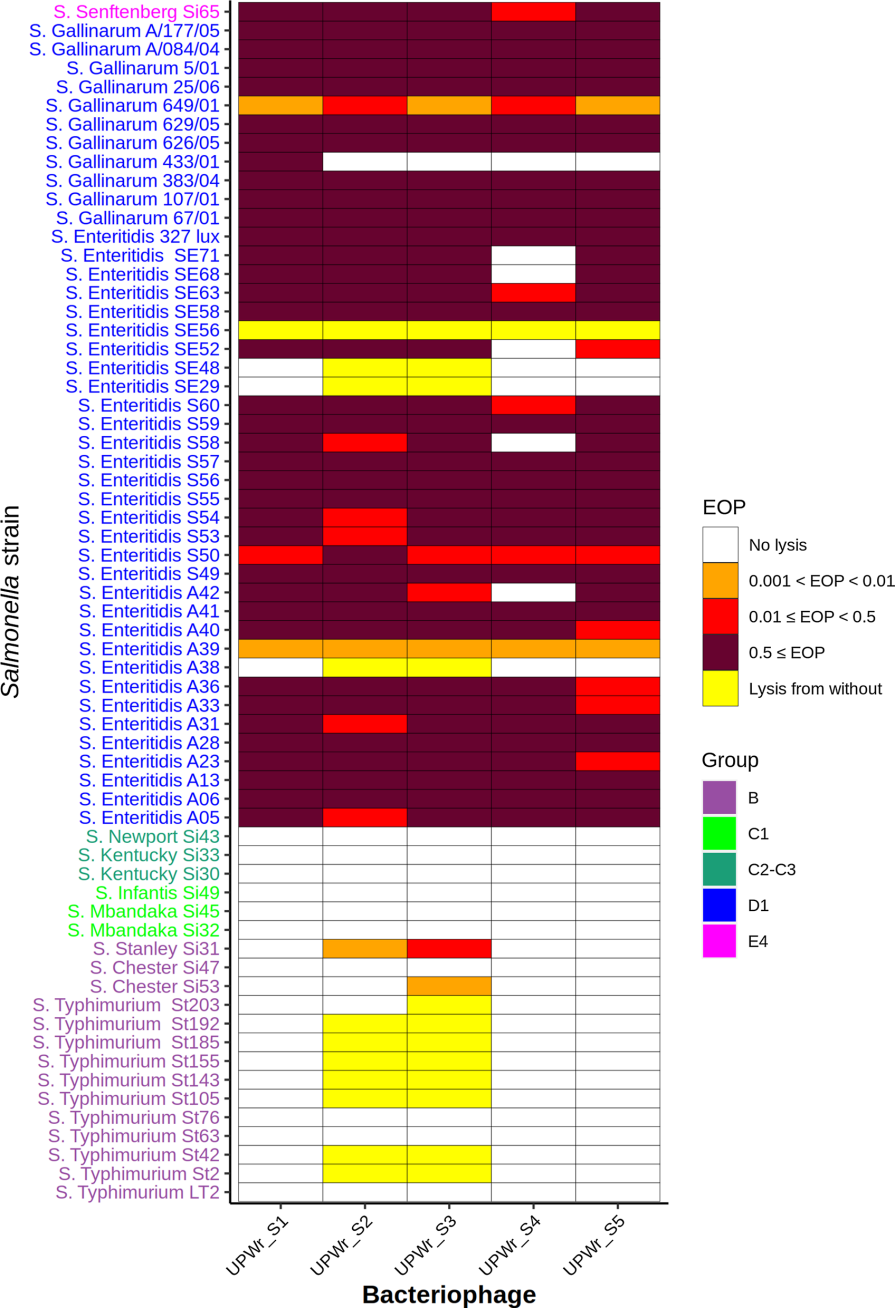
Bacteriophage host range and EOP. Bacteriophages were tested for host ranges and EOP against 64 *Salmonella* strains belonging to 10 serovars. Obtained results were rearranged using hierarchical clustering analysis and plotted as a heatmap. Bacteriophages used in this analysis are presented on the X axis and the *Salmonella* strains are listed on the Y axis. Each rectangle shows the lytic effect of one bacteriophage to one host and the colors correspond to the lysis pattern: purple, EOP ≥ 0.5; red, 0.01 ≤ EOP < 0.5; orange, 0.0001 < EOP < 0.01; yellow, lysis from without; white, no lysis

A total of 161 isolated phages, named collectively UPWr_S, were tested against 64 *Salmonella* strains representing 10 different *S. enterica* serovars. Five phages with strong lytic activity and infecting the largest number of *Salmonella* strains compared to the rest of the analyzed phages, named UPWr_S1-5, were chosen for further studies (Fig. 1). Phages UPWr_S2 and UPWr_S3 lysed 51 and 53 *S*. *enterica* strains, respectively. Bacteriophage UPWr_S3 was effective against all 30 analyzed *S.* Enteritidis strains, with 24 strains highly sensitive to phage infection (EOP ≥ 0.5). 8/11 *S.* Typhimurium strains (73%) were lysed in the process of lysis from without, as were 10/11 *S.* Gallinarum strains (91%), with 9 strains being infected with high effectiveness (EOP ≥ 0.5). This phage was able to lyse representatives of *S.* Senftenberg (EOP ≥ 0.5), *S*. Chester and *S.* Stanley (0.0001 < EOP < 0.01). The phage UPWr_S3 did not infect *S.* Kentucky, *S.* Mbandaka, *S.* Infantis or *S.* Newport. UPWr_S2 exhibited a similar host range profile, with a decreased ability to lyse *S*. Typhimurium strains (7/11) in the lysis from without process, and an inability to infect *S*. Chester, *S*. Infantis, *S*. Newport, *S*. Kentucky or *S*. Mbandaka. UPWr_S1 was the only UPWr_S phage which in addition to the majority of *S.* Enteritidis strains (81% with EOP ≥ 0.5) infected all tested *S*. Gallinarum strains (10 with EOP ≥ 0.5) and *S*. Senftenberg with EOP ≥ 0.5, and did not exhibit lytic activity against strains belonging to the remaining serovars. The phage UPWr_S5 showed lytic activity similar to UPWr_S1, and infected 64% of *S.* Enteritidis and 88% of *S*. Gallinarum strains with EOP ≥ 0.5. Finally, the phage UPWr_S4 lysed with high effectiveness (EOP ≥ 0.5) 58% of *S*. Enteritidis strains and 88% of *S.* Gallinarum strains. Similar to other characterized phages, UPWr_S4 was able to infect the *S.* Senftenberg strain (EOP ≥ 0.5) but did not lyse *S.* Typhimurium strains or strains from the rest of the analyzed serovars.

Taken together, all UPWr_S phages showed a high ability to infect the majority of tested *Salmonella* strains belonging to Enteritidis and Gallinarum serovars, and all of them infected *S.* Senftenberg. Only the phages UPWr_S2 and UPWr_S3 could lyse the majority of strains belonging to *S. *Typhimurium nonspecifically, utilizing the lysis from without mechanism, and *S.* Stanley. *S.* Chester strains were lysed only by UPWr_S3.

### Assessment of life cycle and genome analysis revealed the lytic infection cycle

Chemical treatment of lysogenic strains with mitomycin C is known to cause induction of prophages (Owen et al., 2017). All UPWr_S phages produce clear plaques on their *Salmonella* hosts (Additional file 3: Fig. S1). In order to show that UPWr_S1-5 phages undergo the lytic cycle, induction of *Salmonella* host strains with mitomycin C was performed. It was found that all analyzed UPWr_S phage genomes did not contain any mitomycin C-inducible prophages and develop the lytic life cycle. As the positive control, *S*. Typhimurium LT2 colonies resistant to lysogenic phage P22 were treated with mitomycin C, yielding plaques with a turbid center at a titer of about 10^7^ PFU/ml, indicating effective prophage induction. In contrast, *S*. Typhimurium LT2 colonies resistant to strictly lytic Felix O1 treated with mitomycin C did not form plaques, indicating the lack of inducible prophages. Moreover, genome analysis revealed that UPWr_S1-5 phage genomes did not encode known integrase or excisionase; hence it is likely that these phages proceed through the lytic life cycle.

### Phage morphology

**Fig. 2:**
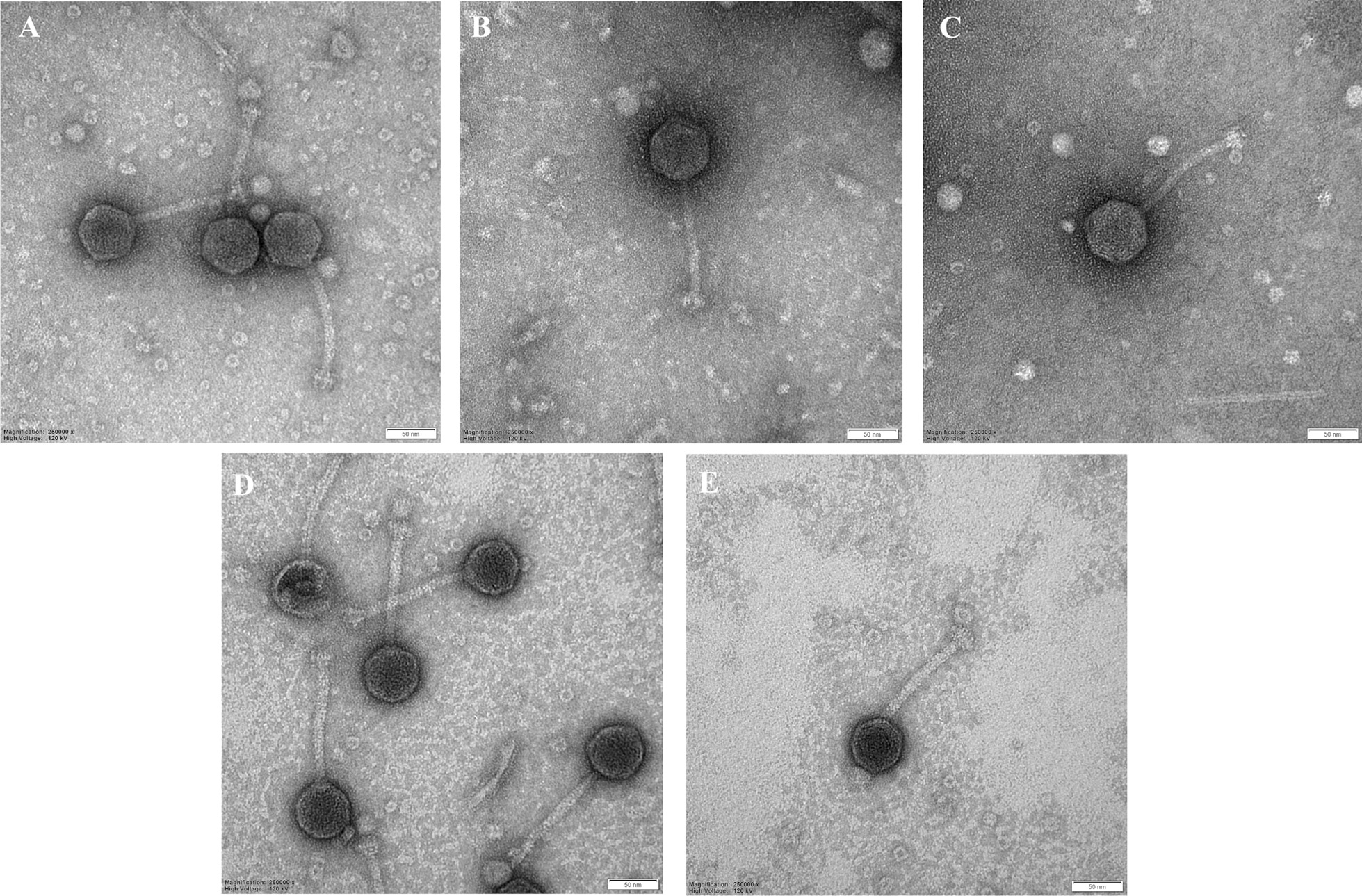
Transmission electron microscopic (TEM) analysis of UPWr_S phages. All five UPWr_S phages were identified by TEM, UPWr_S1 (**a**), UPWr_S2 (**b**), UPWr_S3 (**c**), UPWr_S4 (**d**) and UPWr_S5 (**e**). The black bar represents 50 nm

To classify the *Salmonella* UPWr_S1-5 phages into morphotype-specific groups, transmission electron microscopy (TEM) was employed and *Salmonella* bacteriophage particles were examined at 250,000 magnification. All five UPWr_S phages exhibited the B1 morphotype with isometric capsids (ca. 50–57 nm), long tails (length: ca. 112–124 nm) and clearly visible tail fibers (Fig. 2). Morphological features are described in Table 1. Morphological analysis of phages showed that they can be classified as members of the genus *Jerseyvirus* within the *Siphoviridae* family.

**Table 1:** UPWr_S phages’ morphological features, genome size and biological characteristics

	UPWr_S1	UPWr_S2	UPWr_S3	UPWr_S4	UPWr_S5
Capsid size, nm ± SE^*^	52 ± 6	57 ± 4	52 ± 5	56 ± 4.5	51 ± 4
Tail size, nm ± SE	134 ± 2	136 ± 7.1	138 ± 5	129 ± 4	131 ± 3.6
Genome size, bp	44 417	44 225	44 154	44 330	44 548
GC content, %	49.85	50.02	50.01	50.02	49.99
MOI	1	0.1	0.1	1	1
Latent period, min	15	12	9	24	26
Burst size, PFU/cell	201	89	92	48	23
Adsorption degree^**^	90	93	99	80	92

UPWr_S phages’ morphological and biological characterization revealed their high degree of similarity. Calculated latent period and burst size for each phage showed the shorter latent period with a higher burst size and lower MOI of phages UPWr_S2 and UPWr_S3

^*^Head diameter is calculated for isometric capsids. All measurements were made with the program ImageJ. 35 particles were measured for each phage and standard deviation was calculated (± SD)

^**^Phage particles adsorbed within 10 min

### One-step growth curve bacteriophages

One-step growth curve analysis of UPWr_S phages was performed to determine the latent period and relative burst size per infected bacterial cell. Data generated were analyzed and used to construct the one-step growth curve (Additional file 4: Fig. S2). The latent period of UPWr_S1 was assigned to be 15 min, whereas for phages UPWr_S2 and UPWr_S3 calculated latent periods were 12 and 9 min, respectively. UPWr_S4 and UPWr_S5 phages had the longest latent periods, 24 and 25 min, respectively (Table 1). The phage UPWr_S1 had the largest burst size per infected bacterium (201 PFU), while the phages UPWr_S2, UPWr_S3 and UPWr_S5 had a smaller burst size (89, 92 and 92 PFU, respectively), and UPWr_S4 had the smallest burst size per infected bacterium (60 PFU).

### Determination of adsorption rate

Adsorption studies were performed to identify the adsorption rate of UPWr_S phages on host bacteria. According to the phage adsorption assay, 90, 93, 99, 80 and 92% of UPWr_S1, UPWr_S2, UPWr_S3, UPWr_S4 and UPWr_S5, respectively, could adsorb to host bacteria within 10 min (Table 1), indicating that the phages were readily adsorbed to the host (Additional file 5: Fig. S3).

### Characterization of UPWr_S phage genomes

UPWr_S1-5 phage genomes were sequenced and analyzed on the one hand for their relatedness to each other and, on the other hand, for the presence of any differences. Genome sequencing generated 271,976–408,810 reads for five novel phages with around 1000 × coverage. Analyzed genomes ranged in size from approximately 42 to 43 kb with G + C contents of about 50%. Fifty-one genes, called core genes, were found in all UPWr_S phage genomes, while 19 were not present in all genomes and are called accessory genes. Among them, 14 were of unknown function (Additional file 6: Table S2).

Functional analysis revealed that 14 core genes, present in all 5 genomes, were involved in morphogenesis, coding for capsid, neck, tail fiber, tailspike, capsid decoration and putative tail tape measure proteins. Among accessory genes with predicted morphogenetic function, one gene present in the UPWr_S1 phage genome coded for a fragment of putative head–tail joining protein (gp27) and another gene found in the UPWr_S5 phage genome encoded a fragment of a tail fiber protein (gp70). All of the UPWr_S phage genomes possessed eight core genes responsible for phage replication such as DNA polymerase, recombination endonuclease, putative homing endonuclease, inteins and large and small terminase subunits. Further analysis revealed the presence of three accessory genes involved in phage replication. The genomes of UPWr_S2, UPWr_S3 and UPWr_S4 phages shared one accessory gene encoding a putative protein with homology to homing endonuclease (gp63). In UPWr_S1 and UPWr_S5 phage genomes, DNA primase (gp46) was found. In the genome of the UPWr_S5 phage a gene coding for DNA-cytosine methylase (gp67) taking part in protection from host-encoded exonucleases during phage DNA ejection was found. All UPWr_S phage genomes contained a mobile element such as the putative intein-containing capsid morphogenesis protein (gp15). Another mobile element such as homing endonuclease (gp63) was revealed in UPWr_S2, UPWr_S3 and UPWr_S4 phage genomes.

Three regulatory core genes, the gene coding for helix-turn-helix transcriptional regulator (gp58), the gene coding for XRE family transcriptional regulator (gp55), and the gene coding for putative DNA-binding protein (gp31), were detected. Finally, genes facilitating host lysis such as putative lysozyme and holin class II (gp06) and one superinfection immunity protein (gp35) were displayed within core genes and present in all UPWr_S phage genomes. In all UPWr_S genomes, we found the gene encoding enolase (gp40) with unknown function in phages. Our analysis indicated that UPWr_S phages were deprived of known genes encoding toxins, antibiotic resistance or virulence. Also, UPWr_S phage genomes did not contain tRNA genes (Additional file 6: Table S2). Notably, 23 genes representing a significant proportion of core genes were of unknown function.

**Fig. 3:**
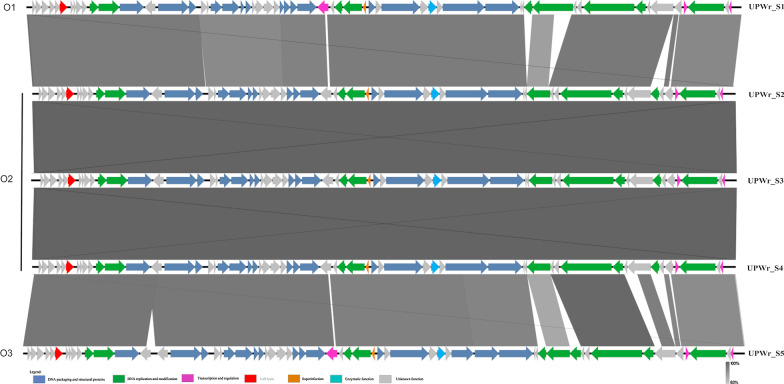
Comparison of UPWr_S1-5 phage genomes’ organization. Whole genome sequence comparisons of UPWr_S1-5 bacteriophages were generated with BLASTn and visualized with EasyFig software. Predicted genes are indicated by arrows and are color coded by putative function. Relatedness between marked regions is presented by percent similarity (grayscale). Colors correspond to the functional protein group: navy blue—DNA packaging and structural proteins; green—DNA replication and modification; pink—transcription and regulation; red—cell lysis; orange—superinfection exclusion; light blue—enzymatic function; gray—unknown function

Sequence comparison allowed for clear discrimination of 3 kinds of genomes named O1, O2 and O3 (Fig. 3). Genomes of phages UPWr_S1 and UPWr_S5 represented O1 and O3, respectively, while UPWr_S2, UPWr_S3 and UPWr_S4 phage genomes all represented the O2 type.

### Phylogenetic analysis revealed close relatedness of UPWr_S phages to the genus *Jerseyvirus*

**Fig. 4:**
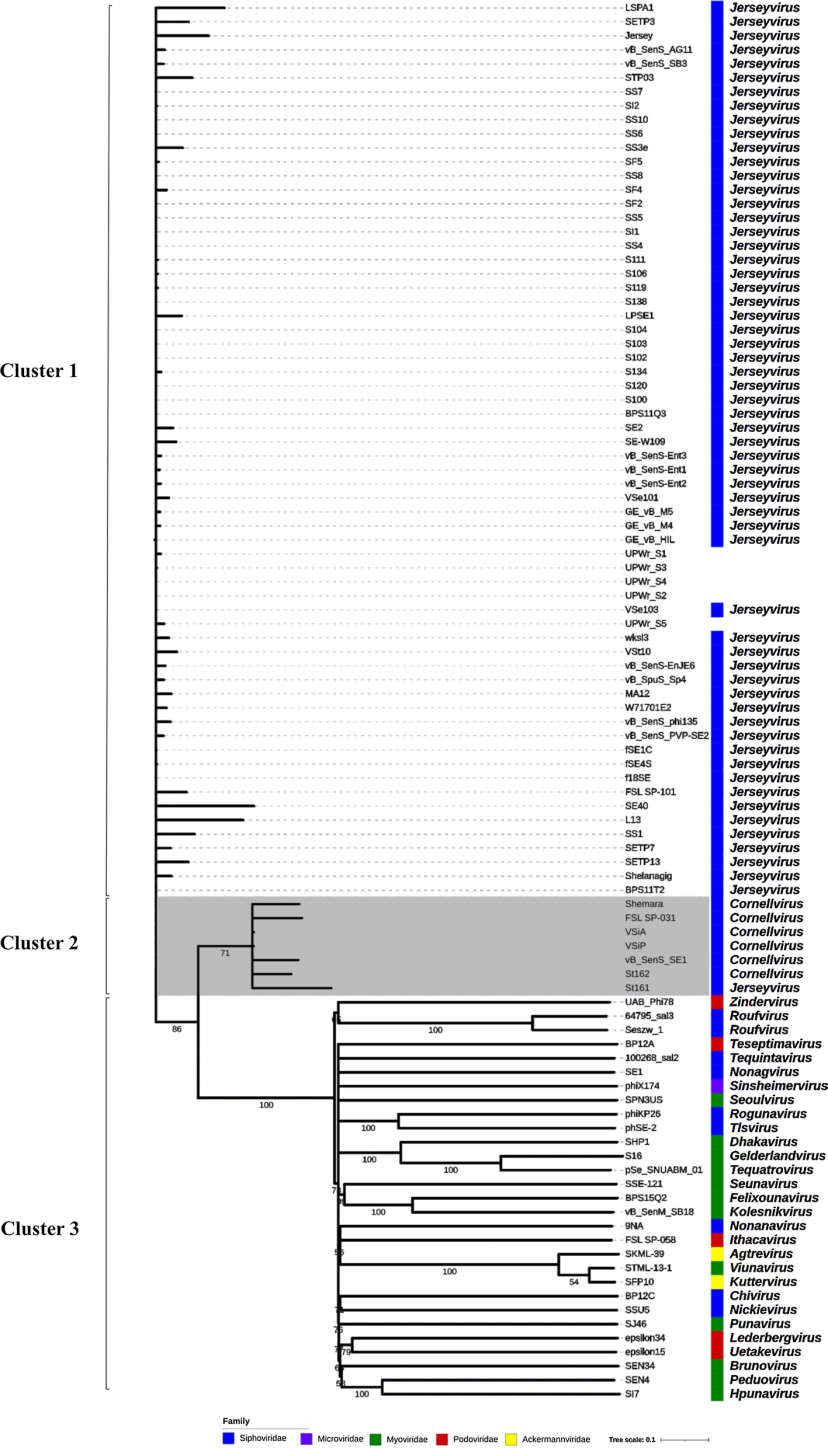
Phylogenetic tree of *Salmonella* bacteriophages. A set of genomes representing available bacteriophages belonging to the genus *Jerseyvirus* and one representative genome from other genera infecting *Salmonella* were selected from the GenBank Virus database. All pairwise comparisons of the nucleotide sequences were conducted using the Genome-BLAST Distance Phylogeny (GBDP) method under settings recommended for prokaryotic viruses using VICTOR software. The resulting intergenomic distances were used to infer a balanced minimum evolution tree with branch support via FASTME including SPR postprocessing. Branch support was inferred from 100 pseudo-bootstrap replicates each. Branches with bootstrap values below 50 were collapsed and the bootstrap values equal to or above 50 are shown on the remainder of the tree branches. Information about genus and family for each sequence was added using iTOL

To study the evolutionary relationship of UPWr_S phages, their genomes were compared with previously sequenced *Salmonella* phages deposited in GenBank. Sixty phages belonging to the *Jerseyvirus* genus and 35 representatives of other *Salmonella* phage genera were selected and phylogenetic relationship assessment using VICTOR software was performed. Phylogenetic tree analysis identified three distinct clusters of genomes (Fig. 4). Phage genomes with a high level of genetic similarity belonged to clusters 1 and 2 (> 50% bootstrap support). With the exception of the phage St161 (MF158036), all analyzed *Jerseyvirus* phages belonged to cluster 1. Cluster 2 was formed by phages belonging to the genus *Cornellvirus* and phage St161, classified previously as *Jerseyvirus*; cluster 3 comprised phages representing genera belonging to different families and subfamilies. In contrast to clusters 1 and 2, genomes of phages belonging to cluster 3 were characterized by low relatedness.

The analysis of UPWr_S phage genomes revealed that they belonged to cluster 1 and exhibited close relatedness to phages classified as *Jerseyvirus.* ANI of phages belonging to cluster 1 was calculated to be 91%. On the basis of 95% DNA sequence identity [46], UPWr_S phages were categorized and assigned to the genus *Jerseyvirus* (according to ICTV Taxonomy Release #35: 2019) [47], which includes such well-characterized phages as SS3e (AY730274), SE2 (JQ007353), wksl3 (JX202565), vB_SenS_Ent2 (NC_023608), vB_SenS_Ent1 (NC_019539) and vB_SenS_Ent3 (NC_024204), among others [48–52]. UPWr_S phages showed relatedness with *Cornellvirus* phages from cluster 2 assigned to be approximately 73% and were completely unrelated to those from cluster 3, which was confirmed in whole-genome dot plot comparison (Additional file 7: Fig. S4). In this analysis, it was shown that UPWr_S genomes displayed no sequence similarities to phages not belonging to *Guernseyvirinae* subfamilies. These results are consistent with previous findings that Jersey phages show low sequence similarity to phages that are not in the *Siphoviridae* family [53].

Whole genome alignment revealed the presence of 4 differences in nucleotide sequences between UPWr_S2 and UPWr_S3, 8 differences in nucleotide sequences between UPWr_S2 and UPWr_S4, and 5 differences in nucleotide sequences between UPWr_S3 and UPWr_S4. These differences included substitutions and deletions (Table 2). Substitutions resulted in amino acid alterations or single nucleotide changes in intergenic regions.

**Table 2:** Sequence variation between phages UPWr_S2, UPWr_S3 and UPWr_S4

Gene product	Protein function	UPWr_S2 vs UPWr_S3	UPWr_S2 vs UPWr_S4	UPWr_S3 vs UPWr_S4
gp16	Putative head decoration protein	–	R132G^*^	R132G
gp20	Putative major capsid protein	–	V338A	V338A
Region between gp20-21		–	Substitution of *g* to *a*	–
gp25	Putative protein	R185W	R185W	–
gp30	Putative tail protein	A94V	–	V94A
Region between gp30-gp31		Deletion of 1 t^**^ in UPWr_S2	Deletion of 1 *t* in UPWr_S2	–
gp42	Tail fiber protein	Deletion of 6 AA in UPWr_S2 374–379 (MYKDNG)	Deletion of 6 AA in UPWr_S2 374–379 (MYKDNG)	–
gp43	Tailspike	–	S2P, G464D	S2P, G464D

Whole genome alignment revealed the presence of a few differences in nucleotide sequences between UPWr_S2, UPWr_S3 and UPWr_S4 phages. These differences included deletions and substitutions resulting in amino acid alterations or single nucleotide changes in intergenic regions. There are 4 common substitutions between these phages located in predicted head decoration (gp16), putative major capsid protein (gp20) and tailspike protein with endorhamnosidase function (gp43)

^*^Letters corresponding to amino acids are written with capital letters

^**^Letters corresponding to nucleotides are lower case and italicized

– no changes

## Discussion

There is a great interest in various practical applications of bacteriophages against *Salmonella*, with the most attention (and most regulatory approvals) focused on their use to improve food safety [54]. They have been proposed as alternatives to antibiotics in animal health, as biopreservatives in food and as tools for detecting pathogenic bacteria throughout the food chain [55, 56]. Due to the rising numbers of antibiotic-resistant *Salmonella* strains, bacteriophage therapy appears to be one of the most promising tools combating these pathogens [57]. The success of such treatment is largely dependent on the biological properties of the phages being used. As *Salmonella* consists of more than 2,500 serovars, infection efficacy and a wide host spectrum are indispensable requirements for *Salmonella*-targeting phages, and therefore such phages are naturally the most attractive candidates for the treatment of bacterial infections [58]. Temperate bacteriophages exhibit the potential for gene transduction and may be involved in increasing bacterial virulence [59]. Therefore, phages used for environmental, industrial, or medical purposes should undergo only the lytic life cycle to exclude the possibility of horizontal virulence gene transfer [60]. To meet these requirements, five *Salmonella* bacteriophages, named UPWr_S1-5, were selected and characterized according to their host range and life cycle.

It was found that UPWr_S1-5 phages belong to the genus *Jerseyvirus* within the *Siphoviridae* family and share similar G + C and gene content, genome organization and morphology with previously described phages form this genus [48–53]. Members of the genus *Jerseyvirus* are tailed bacteriophages and infect a number of *Salmonella* serovars [50–52], with widespread distribution around the world [50] and common isolation from the environment, the most prominent sources being wastewaters [53]. These phages have recently been approved as a safe anti-*Salmonella* zootechnical additive in water for drinking and liquid complementary feed for all avian species known as BAFASAL [61]. In line with these findings, it was observed that UPWr_S1-5 phages infected 3 to 5 *Salmonella* serovars, including the majority of analyzed *S*. Enteritidis and *S*. Gallinarum clinical strains as well as the frequently isolated *S*. Senftenberg, *S.* Stanley and *S.* Chester strains, which makes them similar to other lytic phages considered to be potential anti-*Salmonella* agents [17, 62]. Previously, lytic bacteriophages with such a host range were deemed to be suitable anti-*Salmonella* control agents [18, 51, 63].

All known members of the proposed genus *Jerseyvirus* are strictly lytic [48, 51, 64]. Therefore, the lytic life cycle of UPWr_S phages was confirmed using the mitomycin C test assay. It should be mentioned that although some viable *Salmonella* phages are not inducible using standard techniques including mitomycin C treatment [65], this induction assay remains an important component of studies aimed at characterization of newly isolated phages. The mitomycin C experimental data were supported by the absence in UPWr_S1-5 phage genomes (similar to other Jersey phages) of *int* and *xis* genes coding for integrase and excisionase, respectively, which often play a role in the establishment of lysogeny. Interestingly, UPWr_S1-5 phage genomes contain the gene *imm* encoding superinfection protein involved in the prevention of infection of already-infected bacteria by other phages [66]. All *Jerseyvirus* phages contain this gene in their genomes and remain lytic, but the mechanism of this phenomenon remains unknown [51, 67].

Another feature of UPWr_S1-5 phages shared with other phages belonging to the genus *Jerseyvirus* and family *Siphoviridae* is the mosaic structure of their genomes and presence of mobile elements [49]. Analysis of UPWr phage genes showed the presence of genes coding for an intein and homing endonucleases, which are functionally associated with this phenomenon. Inteins are known to promote the exchange of flanking genes between related *Salmonella* phages [68], whereas homing endonucleases are DNA-cleaving enzymes that assemble their own reading frames [69], and their activity may lead to mosaicism [70]. It was found that the phages UPWr_S2, UPWr_S3 and UPWr_S4, despite very high genome sequence identity (> 99.9%) and organization, have slightly different functional characteristics. UPWr_S2 and UPWr_S3 exhibited a shorter latent period and larger burst size than UPWr_S4 and other Jersey phages such as Ent1 [50] and wksl3 [51]. As these parameters play an important role in the host lysis system [71], it suggests that UPWr_S2 and UPWr_S3 can be considered as useful anti-*Salmonella* agents.

## Conclusions

In this study, we isolated and characterized five novel UPWr_S1-5 bacteriophages, which were classified in the genus *Jerseyvirus* within the *Siphoviridae* family. UPWr_S1-5 phages infected gastroenteritis-causing *S.* Enteritidis and the etiological factor of fowl typhoid, *S*. Gallinarum. Therefore, because of their ability to infect various *Salmonella* serovars and lytic life cycle, they can be considered as useful tools in biological control of salmonellosis.

### Patents

The phages are part of a Wrocław University of Environmental and Life Sciences patent pending. Poland Patent Application P.430168.

## Supplementary Information

**Additional file 1**:** File S1**. R code to analyse and plot UPWr_S phages host ranges and EOP and construct a heatmap. For host range determination 64* Salmonella* strains belonging to 10 serovars were tested. UPWr_S phages showed a high ability to infect the majority of tested * Salmonella* strains belonging to Enteritidis and Gallinarum serovars, and all of them infected *S*. Senftenberg. Only the phages UPWr_S2 and UPWr_S3 could lyse the majority of strains belonging to *S*. Typhimurium nonspecifically, utilizing the lysis from without mechanism, and *S*. Stanley. *S*. Chester strains were lysed only by UPWr_S3.

**Additional file 2**.** Table S1**. List of phages used in a comparison study to construct the phylogenetic tree in Fig. 4 and their order in the phylogenetic tree. For evolutionary relationships, 60 available genomes of bacteriophages belonging to* Jerseyvirus* and six available genomes of* Cornellvirus* bacteriophages, both members of the* Guernseyvirinae* subfamily,* Siphoviridae* family, and 29 representative genomes from other phage genera infecting *Salmonella*, were selected from the GenBank Virus database.

**Additional file 3**.** Fig. S1**: UPWr_S1 (**a**), UPWr_S2 (**b**), UPWr_S3 (**c**), UPWr_S4 (**d**) and UPWr_S5 (**e**) phages’ plaque morphology on* Salmonella* Enteritidis lawn. Analysis of plaque morphology revealed that the plaque morphology of each phage was similar, with medium size and a light halo around them. Diameters of plaques were measured manually and diameters of plaques for phages UPWr_S1, UPWr_S2, UPWr_S3, UPWr_S4 and UPWr_S5 were 1.04 mm +/− 0.28 mm, 1.71 mm +/− 0.21 mm, 1.72 mm +/− 0.26 mm, 1.58 mm +/− 0.32 mm, 2.81 mm +/− 0.32 mm, respectively.

**Additional file 4**.** Fig. S2**: One-step growth curves of UPWr_S phages. The one-step growth curve of UPWr_S phages propagated on their respective hosts in LB medium revealed that the latent periods and burst sizes were approximately 15, 12, 9, 24, 23 and 92 minutes for phages (**a**) UPWr_S1, (**b**) UPWr_S2, (**c**) UPWr_S3, (**d**) UPWr_S4 and (**e**) UPWr_S5, respectively. The average burst size was estimated to be 201, 89, 92, 48, 92 PFU/cell for phages UPWr_S1, UPWr_S2, UPWr_S3, UPWr_S4 and UPWr_S5, respectively.

**Additional file 5**.** Fig. S3**: Adsorption curves of UPWr_S phages. Adsorption assays showed that the adsorption rates within 10 minutes for phages (**a**) UPWr_S1, (**b**) UPWr_S2, (**c**) UPWr_S3, (**d**) UPWr_S4 and (**e**) UPWr_S5 were 90, 93, 99, 80 and 92%, respectively.

**Additional file 6**.** Table S2**: Predicted ORFs and genes encoded by the UPWr_S phage genomes. Protein sequences of the predicted ORFs of UPWr_S phages were subjected to the BLASTp program to analyze their best known matches on the NCBI website (https://blast.ncbi.nlm.nih.gov). The average nucleotide identity and query coverage were calculated by BLASTp with the cutoff E-value set at 1E-04. “Related phages” refers to a top hit from NCBI BLASTp.

**Additional file 7**.** Fig. S4**: Polydot plot comparison of newly sequenced genomes with selected* Salmonella* phages. All-against-all genome sequence dot plot comparisons of UPWr_S1-5 bacteriophages with selected * Salmonella* phages belonging to clusters 2 and 3 were performed using FlexiDot. When the DNA residues of both sequences match at the same location on the plot, a dot is drawn at the corresponding position. Once the dots have been plotted, they will combine to form lines that correspond to similar fragments of the genomes. On the main diagonal the sequence’s alignment with itself is presented.

## Data Availability

The annotated UPWr_S phage genome sequences were deposited in the NCBI database at [72] under accession numbers MT588083, MT632017, MT632018, MT632019 and MT632020 for UPWr_S1, UPWr_S2, UPWr_S3, UPWr_S4 and UPWr_S5, respectively.

## Abbreviations

ANI: Average nucleotide identity
GBDP: Genome-BLAST Distance Phylogeny
DSMZ: German Collection of Microorganisms and Cell Cultures GmbH
NCBI: National Center for Biotechnology Information
LB: Luria–Bertani
ORF: Open reading frame
PFU: Plaque forming unit
TEM: Transmission electron microscopy

## References

[1] Kuźmińska-Bajor M, Kuczkowski M, Grzymajło K, Wojciech Ł, Sabat M, Kisiela D, et al. Decreased colonization of chicks by Salmonella enterica serovar Gallinarum expressing mannose-sensitive FimH adhesin from Salmonella enterica serovar Enteritidis. Vet Microbiol. 2012;158. 10.1016/j.vetmic.2012.01.029 22364838

[2] Gayet R, Bioley G, Rochereau N, Paul S, Corthésy B. Vaccination against salmonella infection: the mucosal way. Microbiol Mol Biol Rev. 2017;81. 10.1128/MMBR.00007-17 PMC5584317 28615285

[3] Grzymajlo K, Ugorski M, Suchanski J, Kedzierska AE, Kolenda R, Jarzab A, et al. The Novel Type 1 Fimbriae FimH Receptor Calreticulin Plays a Role in Salmonella Host Specificity. Front Cell Infect Microbiol. 2017;7. 10.3389/fcimb.2017.00326 PMC5516122 28770174

[4] Antunes P, Mourão J, Campos J, Peixe L. Salmonellosis: the role of poultry meat. Clin Microbiol Infect. 2016;22. 10.1016/j.cmi.2015.12.004 26708671

[5] Shivaning Karabasanavar N, Benakabhat Madhavaprasad C, Agalagandi Gopalakrishna S, Hiremath J, Shivanagowda Patil G, B Barbuddhe S. Prevalence of Salmonella serotypes S. Enteritidis and S. Typhimurium in poultry and poultry products. J Food Saf. 2020;40:e12852.

[6] Shivaprasad HL. Fowl typhoid and pullorum disease. Rev Sci Tech l’OIE. 2000;19. 10.20506/rst.19.2.1222 10935271

[7] Xiong W, Wang Y, Sun Y, Ma L, Zeng Q, Jiang X, et al. Antibiotic-mediated changes in the fecal microbiome of broiler chickens define the incidence of antibiotic resistance genes. Microbiome. 2018;6. 10.1186/s40168-018-0419-2 PMC5811963 29439741

[8] Cohen E, Davidovich M, Rokney A, Valinsky L, Rahav G, Gal‐Mor O. Emergence of new variants of antibiotic resistance genomic islands among multidrug‐resistant *Salmonella enterica* in poultry. Environ Microbiol. 2020;22. 10.1111/1462-2920.14858 31715658

[9] Romero-Calle D, Guimarães Benevides R, Góes-Neto A, Billington C. Bacteriophages as Alternatives to Antibiotics in Clinical Care. Antibiotics. 2019;8. 10.3390/antibiotics8030138 PMC6784059 31487893

[10] Lewis R, Hill C. Overcoming barriers to phage application in food and feed. Curr Opin Biotechnol. 2020;61. 10.1016/j.copbio.2019.09.018 31726332

[11] Huh H, Wong S, St. Jean J, Slavcev R. Bacteriophage interactions with mammalian tissue: Therapeutic applications. Adv Drug Deliv Rev. 2019;145. 10.1016/j.addr.2019.01.003 30659855

[12] Malik DJ, Sokolov IJ, Vinner GK, Mancuso F, Cinquerrui S, Vladisavljevic GT, et al. Formulation, stabilisation and encapsulation of bacteriophage for phage therapy. Adv Colloid Interface Sci. 2017;249. 10.1016/j.cis.2017.05.014 28688779

[13] Principi N, Silvestri E, Esposito S. Advantages and Limitations of Bacteriophages for the Treatment of Bacterial Infections. Front Pharmacol. 2019;10. 10.3389/fphar.2019.00513 PMC6517696 31139086

[14] Ackermann HW, Petrow S, Kasatiya SS. Unusual bacteriophages in Salmonella newport. J Virol. 1974;13. 10.1128/jvi.13.3.706-711.1974 PMC355357 4132670

[15] Sevilla-Navarro S, Catalá-Gregori P, Marin C. Salmonella Bacteriophage Diversity According to Most Prevalent Salmonella Serovars in Layer and Broiler Poultry Farms from Eastern Spain. Animals. 2020;10. 10.3390/ani10091456 PMC7552790 32825110

[16] Hyman P, Abedon ST. Bacteriophage Host Range and Bacterial Resistance. 2010. 10.1016/S0065-2164(10)70007-1 20359459

[17] Li M, Lin H, Jing Y, Wang J. Broad-host-range Salmonella bacteriophage STP4-a and its potential application evaluation in poultry industry. Poult Sci. 2020;99. 10.1016/j.psj.2020.03.051 PMC7597861 32616261

[18] Thanki AM, Brown N, Millard AD, Clokie MRJ. Genomic characterization of jumbo Salmonella phages that effectively target United Kingdom pig-associated Salmonella serotypes. Front Microbiol. 2019. 10.3389/fmicb.2019.01491 PMC6614189 31312191

[19] Bielke L, Higgins S, Donoghue A, Donoghue D, Hargis BM. Salmonella Host Range of Bacteriophages That Infect Multiple Genera. Poult Sci. 2007;86. 10.3382/ps.2007-00250 18029799

[20] Gambino M, Nørgaard Sørensen A, Ahern S, Smyrlis G, Gencay YE, Hendrix H, et al. Phage S144, a New Polyvalent Phage Infecting Salmonella spp. and Cronobacter sakazakii. Int J Mol Sci. 2020;21. 10.3390/ijms21155196 PMC7432712 32707941

[21] Oliveira A, Sillankorva S, Quinta R, Henriques A, Sereno R, Azeredo J. Isolation and characterization of bacteriophages for avian pathogenic *E. coli* strains. J Appl Microbiol. 2009;106. 10.1111/j.1365-2672.2009.04145.x 19239552

[22] Adams MH. Bacteriophages. Citeseer; 1959.

[23] Kutter E. Phage Host Range and Efficiency of Plating. 2009. 10.1007/978-1-60327-164-6_14 19066818

[24] Petsong K, Benjakul S, Chaturongakul S, Switt A, Vongkamjan K. Lysis Profiles of Salmonella Phages on Salmonella Isolates from Various Sources and Efficiency of a Phage Cocktail against S. Enteritidis and S. Typhimurium. Microorganisms. 2019;7. 10.3390/microorganisms7040100 PMC6518243 30959743

[25] Großwendt A, Röglin H. Improved Analysis of Complete-Linkage Clustering. 2015.

[26] Wickham H. ggplot2. New York, NY: Springer New York; 2009.

[27] Owen S V., Wenner N, Canals R, Makumi A, Hammarlöf DL, Gordon MA, et al. Characterization of the prophage repertoire of African Salmonella Typhimurium ST313 reveals high levels of spontaneous induction of novel phage BTP1. Front Microbiol. 2017;8 FEB. 10.3389/fmicb.2017.00235 PMC5322425 28280485

[28] Rahman M, Kim S, Kim SM, Seol SY, Kim J. Characterization of induced *Staphylococcus aureus* bacteriophage SAP-26 and its anti-biofilm activity with rifampicin. Biofouling. 2011;27. 10.1080/08927014.2011.631169 22050201

[29] Yu YP, Gong T, Jost G, Liu WH, Ye DZ, Luo ZH (2013). Isolation and characterization of five lytic bacteriophages infecting a Vibrio strain closely related to Vibrio owensii. FEMS Microbiol Lett.

[30] Hadas H, Einav M, Fishov I, Zaritsky A. Bacteriophage T4 Development Depends on the Physiology of its Host *Escherichia Coli*. Microbiology. 1997;143. 10.1099/00221287-143-1-179 9025292

[31] Wingett SW, Andrews S. FastQ Screen: A tool for multi-genome mapping and quality control. F1000Research. 2018;7. 10.12688/f1000research.15931.1 PMC6124377 30254741

[32] Page AJ, De Silva N, Hunt M, Quail MA, Parkhill J, Harris SR, et al. Robust high-throughput prokaryote de novo assembly and improvement pipeline for Illumina data. Microb Genomics. 2016;2. 10.1099/mgen.0.000083 PMC5320598 28348874

[33] Seemann T. Prokka: rapid prokaryotic genome annotation. Bioinformatics. 2014;30. 10.1093/bioinformatics/btu153 24642063

[34] Pritchard L, Glover RH, Humphris S, Elphinstone JG, Toth IK. Genomics and taxonomy in diagnostics for food security: soft-rotting enterobacterial plant pathogens. Anal Methods. 2016;8.

[35] Sullivan MJ, Petty NK, Beatson SA. Easyfig: a genome comparison visualizer. Bioinformatics. 2011;27. 10.1093/bioinformatics/btr039 PMC3065679 21278367

[36] Nucleotide BLAST: Search nucleotide databases using a nucleotide query. https://blast.ncbi.nlm.nih.gov/Blast.cgi?PROGRAM=blastn&PAGE_TYPE=BlastSearch&LINK_LOC=blasthome. Accessed 15 Jul 2021.

[37] Protein BLAST: search protein databases using a protein query. https://blast.ncbi.nlm.nih.gov/Blast.cgi?PROGRAM=blastp&PAGE_TYPE=BlastSearch&LINK_LOC=blasthome. Accessed 15 Jul 2021.

[38] Pfam: Home page. https://pfam.xfam.org/. Accessed 15 Jul 2021.

[39] PHYRE2 Protein Fold Recognition Server. http://www.sbg.bio.ic.ac.uk/phyre2/html/page.cgi?id=index. Accessed 15 Jul 2021.

[40] Kelly CR, Kahn S, Kashyap P, Laine L, Rubin D, Atreja A, et al. Update on fecal microbiota transplantation 2015: indications, methodologies, mechanisms, and outlook. Gastroenterology. 2015;149. 10.1053/j.gastro.2015.05.008 PMC4755303 25982290

[41] ARAGORN detects tRNA, mtRNA and tmRNA genes. http://www.ansikte.se/ARAGORN/. Accessed 15 Jul 2021.

[42] Laslett D. ARAGORN, a program to detect tRNA genes and tmRNA genes in nucleotide sequences. Nucleic Acids Res. 2004;32. 10.1093/nar/gkh152 PMC373265 14704338

[43] iTOL: Interactive Tree Of Life. https://itol.embl.de/. Accessed 15 Jul 2021.

[44] Letunic I, Bork P. Interactive Tree of Life (iTOL) v4: recent updates and new developments. Nucleic Acids Res. 2019;47. 10.1093/nar/gkz239 PMC6602468 30931475

[45] Seibt KM, Schmidt T, Heitkam T. FlexiDot: highly customizable, ambiguity-aware dotplots for visual sequence analyses. Bioinformatics. 2018;34. 10.1093/bioinformatics/bty395 29762645

[46] Adriaenssens E, Brister JR. How to Name and Classify Your Phage: An Informal Guide. Viruses. 2017;9. 10.3390/v9040070 PMC5408676 28368359

[47] ICTV. https://talk.ictvonline.org/files/master-species-lists/m/msl/9601. Accessed 15 Jul 2021.

[48] Anany H, Switt AIM, De Lappe N, Ackermann H-W, Reynolds DM, Kropinski AM, et al. A proposed new bacteriophage subfamily: “Jerseyvirinae.” Arch Virol. 2015;160. 10.1007/s00705-015-2344-z 25663216

[49] Adriaenssens EM, Edwards R, Nash JHE, Mahadevan P, Seto D, Ackermann H-W, et al. Integration of genomic and proteomic analyses in the classification of the Siphoviridae family. Virology. 2015;477. 10.1016/j.virol.2014.10.016 25466308

[50] Turner D, Hezwani M, Nelson S, Salisbury V, Reynolds D. Characterization of the Salmonella bacteriophage vB_SenS-Ent1. J Gen Virol. 2012;93. 10.1099/vir.0.043331-0 22694898

[51] Kang H-W, Kim J-W, Jung T-S, Woo G-J. wksl3, a New biocontrol agent for Salmonella enterica serovars enteritidis and typhimurium in foods: characterization, application, sequence analysis, and oral acute toxicity study. Appl Environ Microbiol. 2013;79. 10.1128/AEM.02793-12 PMC3592225 23335772

[52] Lu M, Liu H, Lu H, Liu R, Liu X. Characterization and genome analysis of a novel salmonella phage vB_SenS_SE1. Curr Microbiol. 2020;77. 10.1007/s00284-020-01879-7 32086533

[53] Olsen NS, Hendriksen NB, Hansen LH, Kot W. A new high-throughput screening method for phages: enabling crude isolation and fast identification of diverse phages with therapeutic potential. PHAGE. 2020;1. 10.1089/phage.2020.0016 PMC9041460 36147828

[54] Vikram A, Woolston J, Sulakvelidze A. Phage biocontrol applications in food production and processing. Curr Issues Mol Biol. 2021. 10.21775/cimb.040.267 32644048

[55] García P, Martínez B, Obeso JM, Rodríguez A. Bacteriophages and their application in food safety. Lett Appl Microbiol. 2008;47. 10.1111/j.1472-765X.2008.02458.x 19120914

[56] Galié S, García-Gutiérrez C, Miguélez EM, Villar CJ, Lombó F. Biofilms in the food industry: Health Aspects and Control Methods. Front Microbiol. 2018;9. 10.3389/fmicb.2018.00898 PMC5949339 29867809

[57] Gigante A, Atterbury RJ. Veterinary use of bacteriophage therapy in intensively-reared livestock. Virol J. 2019;16. 10.1186/s12985-019-1260-3 PMC6909661 31831017

[58] Ross A, Ward S, Hyman P. More is better: selecting for broad host range bacteriophages. Front Microbiol. 2016;7. 10.3389/fmicb.2016.01352 PMC5014875 27660623

[59] Davies E V., Winstanley C, Fothergill JL, James CE. The role of temperate bacteriophages in bacterial infection. FEMS Microbiol Lett. 2016;363. 10.1093/femsle/fnw015 26825679

[60] Harada LK, Silva EC, Campos WF, Del Fiol FS, Vila M, Dąbrowska K, et al. Biotechnological applications of bacteriophages: State of the art. Microbiol Res. 2018;212–213. 10.1016/j.micres.2018.04.007 29853167

[61] EFSA Panel on Additives, or Substances used in Animal Feed (FEEDAP) P, Bampidis V, Azimonti G, Bastos M de L, Christensen H, et al. Safety and efficacy of a feed additive consisting on the bacteriophages PCM F/00069, PCM F/00070, PCM F/00071 and PCM F/00097 infecting Salmonella Gallinarum B/00111 (Bafasal®) for all avian species (Proteon Pharmaceuticals S.A.). EFSA J. 2021;19:e06534. 10.2903/j.efsa.2021.6534 PMC8127046 34025802

[62] Santos SB, Kropinski AM, Ceyssens P-J, Ackermann H-W, Villegas A, Lavigne R, et al. Genomic and proteomic characterization of the broad-host-range salmonella phage PVP-SE1: creation of a new phage genus. J Virol. 2011;85. 10.1128/JVI.01769-10 PMC3194984 21865376

[63] Santos SB, Fernandes E, Carvalho CM, Sillankorva S, Krylov VN, Pleteneva EA, et al. Selection and characterization of a multivalent *Salmonella* phage and its production in a nonpathogenic *Escherichia coli* strain. Appl Environ Microbiol. 2010;76. 10.1128/AEM.00922-10 PMC2976242 20817806

[64] Tiwari BR, Kim S, Kim J (2012). Complete genomic sequence of salmonella enterica serovar enteritidis phage SE2. J Virol.

[65] Hanna LF, Matthews TD, Dinsdale EA, Hasty D, Edwards RA. Characterization of the ELPhiS prophage from salmonella enterica serovar enteritidis strain LK5. Appl Environ Microbiol. 2012;78. 10.1128/AEM.07241-11 PMC3298174 22247173

[66] Berngruber TW, Weissing FJ, Gandon S. Inhibition of superinfection and the evolution of viral latency. J Virol. 2010;84. 10.1128/JVI.00865-10 PMC2937782 20660193

[67] Sabzali S, Bouzari M. Isolation, identification and some characteristics of two lytic bacteriophages against *Salmonella enterica* serovar Paratyphi B and *S. enterica* serovar Typhimurium from various food sources. FEMS Microbiol Lett. 2021;368. 10.1093/femsle/fnab037 33830213

[68] Pickard D, Thomson NR, Baker S, Wain J, Pardo M, Goulding D, et al. Molecular characterization of the *Salmonella enterica* Serovar Typhi Vi-Typing Bacteriophage E1. J Bacteriol. 2008;190. 10.1128/JB.01654-07 PMC2293211 18192390

[69] Stoddard BL. Homing endonuclease structure and function. Q Rev Biophys. 2006;38. 10.1017/S0033583505004063 16336743

[70] Kwan T, Liu J, DuBow M, Gros P, Pelletier J. The complete genomes and proteomes of 27 Staphylococcus aureus bacteriophages. Proc Natl Acad Sci. 2005;102. 10.1073/pnas.0501140102 PMC556006 15788529

[71] Santos SB, Carvalho C, Azeredo J, Ferreira EC. Population dynamics of a salmonella lytic phage and its host: implications of the host bacterial growth rate in modelling. PLoS One. 2014;9. 10.1371/journal.pone.0102507 PMC4106826 25051248

[72] National Center for Biotechnology Information. https://www.ncbi.nlm.nih.gov/. Accessed 15 Jul 2021.

