# Supplementary material for: Genomic and functional characterization of five novel Salmonella-targeting bacteriophages
Source: Virol J. 2021 Sep 8;18:183. doi: 10.1186/s12985-021-01655-4 (PMC8425127; doi:10.1186/s12985-021-01655-4)
Supplement: Supplementary file 1 — Additional file 1: File S1. R code to analyse and plot UPWr_S phages host ranges and EOP and construct a heatmap. For host range determination 64 Salmonella strains belonging to 10 serovars were tested. UPWr_S phages showed a high ability to infect the majority of tested Salmonella strains belonging to Enteritidis and Gallinarum serovars, and all of them infected S. Senftenberg. Only the phages UPWr_S2 and UPWr_S3 could lyse the majority of strains belonging to S. Typhimurium nonspecifically, utilizing the lysis from without mechanism, and S. Stanley. S. Chester strains were lysed only by UPWr_S3. [file 12985_2021_1655_MOESM1_ESM.pdf]

Additional file 1. File 1. R code to generate figure 1

```
#packages
library("xlsx")
library("tidyverse")
library("ggplot2")
library("reshape2")
library("grid")
library("gridExtra")
library("patchwork")

# upload data

host_lysis_EOP <- read.xlsx("07.07.2021 efficiency of plating.xlsx", 
sheetName = "Arkusz2") %>%
  select(1:6)

#melt the data
host_lysis_EOP.long <- mutate(host_lysis_EOP,
                              Strain=as.character(Strain)) %>%
  melt(id.vars = "Strain") %>%
  mutate(value = as.character(value))


#load the data with serogroups info

Salmonella_serogroup_info <- 
read.xlsx("table_serovars_vs_serogroups_01.11.2020.xlsx", sheetName = 
"Arkusz1") %>%
  mutate(Serogroup = as.factor(Serogroup))

levels(Salmonella_serogroup_info$Serogroup) <-c("E4", "B", "C1", "C2-C3", 
"D1")
Salmonella_serogroup_info$Serogroup <- 
factor(Salmonella_serogroup_info$Serogroup, levels = c( "B", "C1", "C2-C3", 
"D1", "E4"))

# here define colors for the whole plot

label_colors <- c( "B" = "#984ea3" , "C1" = "#00ff00", "C2-C3" = "#1b9e77",
                  "D1" = "#0000ff","E4" = "#ff00ff")

host_lysis_EOP2 <- full_join(host_lysis_EOP, Salmonella_serogroup_info) %>%
  mutate(color = label_colors[Serogroup])

host_lysis_EOP2 <- host_lysis_EOP2[order(host_lysis_EOP2$Serogroup),]
host_lysis_EOP2$Strain

#melt the data
host_lysis_EOP.long2 <- mutate(host_lysis_EOP2,
                          Strain=as.character(Strain)) %>%
  melt(id.vars = c("Strain","Serogroup","color")) %>%
  mutate(value = as.character(value))

host_lysis_EOP.long2[order(host_lysis_EOP.long2$Serogroup),]

# here define colors for the whole plot
label_colors2 <- c("white", "orange", "red", "#630436", "yellow")
```

```
# Heatmap - orginal version with colors on the Y axis


plot_legend <- ggplot(strain_dendrogram_labels, aes(x = group, fill =
group)) +
  geom_bar() +
  scale_fill_manual("Group", values = label_colors,
                    limits = c("B", "C1", "C2-C3", "D1", "E4")) +
  theme(legend.title = element_text(size = 10),
        legend.text = element_text(size = 8))


plot_host_lysis_EOP <- ggplot(data = host_lysis_EOP.long2, aes(x =
variable, y = Strain)) +
  geom_tile(aes(fill = value), color = "black") +
  #coord_equal()+
  scale_fill_manual("EOP", values =label_colors2, labels = c("No lysis",
"0.001 < EOP < 0.01", "0.01 ≤ EOP < 0.5", "0.5 ≤ EOP", "Lysis from
without")) +
  xlab("Bacteriophage") +
  ylab(expression(paste(italic("Salmonella"), " strain")))+
  scale_y_discrete(position = "left", limits = host_lysis_EOP2$Strain) +
 scale_x_discrete(labels = c("UPWr_S1",  "UPWr_S2", "UPWr_S3","UPWr_S4",
"UPWr_S5"))+
  theme_classic()+
  theme(legend.position = "right",
        legend.text = element_text(size = 8),
        legend.title = element_text(size = 10),
        axis.title = element_text(size=12,face="bold"),
        axis.text.x = element_text(size = 8.5, angle = 45, hjust = 1),
        axis.text.y = element_text(size = 9,
                                   color = host_lysis_EOP2[["color"]]))

layout <- c(
  area(t = 1, l = 1, b = 5, r = 4),
  area(t = 2.5, l = 5, b = 5, r = 5)
)
plot_host_lysis_EOP+ get_legend(plot_legend) + plot_layout(design = layout)



tiff(filename = "09.07.2021_host_lysis_EOP_ver6_3.tiff",
     res = 300, units = "mm", width = 240, height = 230)
plot_host_lysis_EOP+  get_legend(plot_legend)
dev.off()
```
