# Supplementary material for: Genomic and functional characterization of five novel Salmonella-targeting bacteriophages
Source: Virol J. 2021 Sep 8;18:183. doi: 10.1186/s12985-021-01655-4 (PMC8425127; doi:10.1186/s12985-021-01655-4)
Supplement: Supplementary file 2 — Additional file 2. Table S1. List of phages used in a comparison study to construct the phylogenetic tree in Fig. 4 and their order in the phylogenetic tree. For evolutionary relationships, 60 available genomes of bacteriophages belonging to Jerseyvirus and six available genomes of Cornellvirus bacteriophages, both members of the Guernseyvirinae subfamily, Siphoviridae family, and 29 representative genomes from other phage genera infecting Salmonella, were selected from the GenBank Virus database. [file 12985_2021_1655_MOESM2_ESM.pdf]

Additional file 2: Table S1. List of phages used in a comparison study to construct the phylogenetic tree.

**No.**	**Phage species**	**Accession number**	**Genus**	**Family**	**Length (bp)**	**Assembly level**
**1**	Salmonella phage BPS11T2	MG646668	*Jerseyvirus*	*Siphoviridae*	43797	complete
**2**	Salmonella phage BPS11Q3	KX405002	*Jerseyvirus*	*Siphoviridae*	43788	complete
**3**	Salmonella phage LPSE1	KY379853	*Jerseyvirus*	*Siphoviridae*	41854	complete
**4**	Salmonella virus SS3e	AY730274	*Jerseyvirus*	*Siphoviridae*	40793	complete
**5**	Salmonella phage SE-W109	KX649889	*Jerseyvirus*	*Siphoviridae*	42147	complete
**6**	Salmonella virus SE2	JQ007353	*Jerseyvirus*	*Siphoviridae*	43221	complete
**7**	Salmonella phage vB_SpuS_Sp4	MH358359	*Jerseyvirus*	*Siphoviridae*	43614	complete
**8**	Salmonella phage vB_SenS-EnJE6	MN336265	*Jerseyvirus*	*Siphoviridae*	43129	complete
**9**	Salmonella virus VSt10	MH424445	*Jerseyvirus*	*Siphoviridae*	41581	complete
**10**	Salmonella virus wksl3	JX202565	*Jerseyvirus*	*Siphoviridae*	42633	complete
**11**	Salmonella phage vB_SenS-Ent2	NC_023608	*Jerseyvirus*	*Siphoviridae*	42093	complete
**12**	Salmonella virus Ent1	NC_019539	*Jerseyvirus*	*Siphoviridae*	42391	complete
**13**	Salmonella phage vB_SenS-Ent3	NC_024204	*Jerseyvirus*	*Siphoviridae*	42764	complete
**14**	Salmonella virus VSe101	MN393078	*Jerseyvirus*	*Siphoviridae*	41757	complete
**15**	Salmonella phage GE_vB_M5	MG969410	*Jerseyvirus*	*Siphoviridae*	44432	complete
**16**	Salmonella phage GE_vB_HIL	MG969408	*Jerseyvirus*	*Siphoviridae*	45334	complete
**17**	Salmonella phage GE_vB_M4	MG969409	*Jerseyvirus*	*Siphoviridae*	44895	complete

**18**	Salmonella phage UPWr/S1	UPWr/S1	*Jerseyvirus*	*Siphoviridae*	43193	complete
**19**	Salmonella phage UPWr/S5	UPWr/S5	*Jerseyvirus*	*Siphoviridae*	43600	complete
**20**	Salmonella virus VSe103	MH424443	*Jerseyvirus*	*Siphoviridae*	42262	complete
**21**	Salmonella phage UPWr/S3	UPWr/S3	*Jerseyvirus*	*Siphoviridae*	42618	complete
**22**	Salmonella phage UPWr/S2	UPWr/S2	*Jerseyvirus*	*Siphoviridae*	42487	complete
**23**	Salmonella phage UPWr/S4	UPWr/S4	*Jerseyvirus*	*Siphoviridae*	42506	complete
**24**	Salmonella phage W71701E2	MN336266	*Jerseyvirus*	*Siphoviridae*	43095	complete
**25**	Salmonella phage MA12	KX245013	*Jerseyvirus*	*Siphoviridae*	41224	complete
**26**	Salmonella phage vB_SenS_phi135	MH992509	*Jerseyvirus*	*Siphoviridae*	43142	complete
**27**	Salmonella phage vB_SenS_PVP-SE2	MF431252	*Jerseyvirus*	*Siphoviridae*	42425	complete
**28**	Salmonella virus f18SE	KT962832	*Jerseyvirus*	*Siphoviridae*	41720	complete
**29**	Salmonella virus f18SE	KR270151	*Jerseyvirus*	*Siphoviridae*	41868	complete
**30**	Salmonella virus f18SE	KT881477	*Jerseyvirus*	*Siphoviridae*	41768	complete
**31**	Salmonella phage Shelanagig	MK931446	*Jerseyvirus*	*Siphoviridae*	42541	complete
**32**	Salmonella virus SETP13	NC_022752	*Jerseyvirus*	*Siphoviridae*	42665	complete
**33**	Salmonella virus SETP3	KF562865	*Jerseyvirus*	*Siphoviridae*	42749	complete
**34**	Salmonella phage S106	MH370363	*Jerseyvirus*	*Siphoviridae*	42976	complete
**35**	Salmonella phage S111	MH370365	*Jerseyvirus*	*Siphoviridae*	43421	complete
**36**	Salmonella phage S134	MH370381	*Jerseyvirus*	*Siphoviridae*	43118	complete

**37**	Salmonella phage S100	MH370358	*Jerseyvirus*	*Siphoviridae*	43468	complete
**38**	Salmonella phage S120	MH370373	*Jerseyvirus*	*Siphoviridae*	43467	complete
**39**	Salmonella phage S102	MH370360	*Jerseyvirus*	*Siphoviridae*	42439	complete
**40**	Salmonella phage S103	MH370361	*Jerseyvirus*	*Siphoviridae*	42441	complete
**41**	Salmonella phage S119	MH370372	*Jerseyvirus*	*Siphoviridae*	43876	complete
**42**	Salmonella phage S104	MH370362	*Jerseyvirus*	*Siphoviridae*	43118	complete
**43**	Salmonella phage S138	MH370384	*Jerseyvirus*	*Siphoviridae*	43119	complete
**44**	Salmonella phage SF2	MK972705	*Jerseyvirus*	*Siphoviridae*	41523	complete
**45**	Salmonella phage SS4	MK761197	*Jerseyvirus*	*Siphoviridae*	41396	complete
**46**	Salmonella phage SI1	MK972691	*Jerseyvirus*	*Siphoviridae*	41396	complete
**47**	Salmonella phage SS5	MK972702	*Jerseyvirus*	*Siphoviridae*	41392	complete
**48**	Salmonella phage SF4	MK761195	*Jerseyvirus*	*Siphoviridae*	43514	complete
**49**	Salmonella phage SS8	MK972706	*Jerseyvirus*	*Siphoviridae*	41772	complete
**50**	Salmonella phage SF5	MK761196	*Jerseyvirus*	*Siphoviridae*	42199	complete
**51**	Salmonella phage SS6	MK972701	*Jerseyvirus*	*Siphoviridae*	41731	complete
**52**	Salmonella phage SS7	MK972703	*Jerseyvirus*	*Siphoviridae*	41795	complete
**53**	Salmonella phage SS10	MK761198	*Jerseyvirus*	*Siphoviridae*	41830	complete
**54**	Salmonella phage SI2	MK761199	*Jerseyvirus*	*Siphoviridae*	42013	complete
**55**	Salmonella virus SETP3	EF177456	*Jerseyvirus*	*Siphoviridae*	42572	complete

**56**	Salmonella virus Jersey	NC_021777	*Jerseyvirus*	*Siphoviridae*	43447	complete
**57**	Salmonella phage STP03	KY176369	*Jerseyvirus*	*Siphoviridae*	43428	complete
**58**	Salmonella phage vB_SenS_SB3	MK578530	*Jerseyvirus*	*Siphoviridae*	41147	complete
**59**	Salmonella virus AG11	JX297445	*Jerseyvirus*	*Siphoviridae*	41546	complete
**60**	Salmonella phage SE40	KY626163	*Jerseyvirus*	*Siphoviridae*	28914	complete
**61**	Salmonella virus SP101	NC_042065	*Jerseyvirus*	*Siphoviridae*	41873	complete
**62**	Salmonella virus LSPA1	KM272358	*Jerseyvirus*	*Siphoviridae*	41880	complete
**63**	Salmonella phage SS1	MK972700	*Jerseyvirus*	*Siphoviridae*	31600	complete
**64**	Salmonella virus L13	KC832325	*Jerseyvirus*	*Siphoviridae*	21248	complete
**65**	Salmonella phage St161	MF158036	*Jerseyvirus*	*Siphoviridae*	29178	complete
**66**	Salmonella phage St162	MF158037	*Cornellvirus*	*Siphoviridae*	42701	complete
**67**	Salmonella phage vB_SenS_SE1	MK479295	*Cornellvirus*	*Siphoviridae*	40987	complete
**68**	Salmonella phage Shemara	MN070121	*Cornellvirus*	*Siphoviridae*	44342	complete
**69**	Salmonella virus SP31	KC139518	*Cornellvirus*	*Siphoviridae*	42215	complete
**70**	Salmonella virus VSiP	MH424444	*Cornellvirus*	*Siphoviridae*	43110	complete
**71**	Salmonella virus VSiA	MN393079	*Cornellvirus*	*Siphoviridae*	42865	complete
**72**	Enterobacteria phage UAB_Phi78	NC_020414	*Zindervirus*	*Podoviridae*	43931	complete
**73**	Salmonella phage Seszw_1	MH791410	*Roufvirus*	*Siphoviridae*	45881	complete
**74**	Salmonella phage 64795_sal3	NC_031918	*Roufvirus*	*Siphoviridae*	45342	complete

**75**	Salmonella virus FSL SP-058	NC_021772	*Ithacavirus*	*Podoviridae*	72394	complete
**76**	Salmonella virus 9NA	NC_025443	*Nonanavirus*	*Siphoviridae*	52869	complete
**77**	Salmonella virus SKML39	NC_019910	*Agtrevirus*	*Ackermannviridae*	159624	complete
**78**	Salmonella virus SFP10	NC_016073	*Kuttervirus*	*Ackermannviridae*	157950	complete
**79**	Salmonella virus STML131	NC_042061	*Viunavirus*	*Myoviridae*	157235	complete
**80**	Salmonella phage SSU5	NC_018843	*Nickievirus*	*Siphoviridae*	103299	complete
**81**	Salmonella phage BP12C	NC_031228	*Chivirus*	*Siphoviridae*	60606	complete
**82**	Salmonella virus SJ46	NC_031129	*Punavirus*	*Myoviridae*	103445	complete
**83**	Salmonella virus Epsilon15	NC_004775	*Uetakevirus*	*Podoviridae*	39672	complete
**84**	Salmonella phage epsilon34	NC_011976	*Lederbergvirus*	*Podoviridae*	43016	complete
**85**	Salmonella virus SEN34	NC_028699	*Brunovirus*	*Myoviridae*	40740	complete
**86**	Salmonella phage SI7	MK972712	*Hpunavirus*	*Myoviridae*	30164	complete
**87**	Salmonella phage SEN4	NC_029015	*Peduovirus*	*Myoviridae*	33509	complete
**88**	Salmonella phage BP12A	NC_031258	*Teseptimavirus*	*Podoviridae*	39696	complete
**89**	Escherichia virus phiX174	HM753716	*Sinsheimervirus*	*Microviridae*	5388	complete
**90**	Salmonella phage phSE-2	NC_031026	*Tlsvirus*	*Siphoviridae*	49167	complete
**91**	Escherichia virus KP26	NC_042038	*Rogunavirus*	*Siphoviridae*	47285	complete
**92**	Salmonella virus SE1Kor	NC_042025	*Nonagvirus*	*Siphoviridae*	60030	complete
**93**	Salmonella virus 118970sal2	NC_031902	*Tequintavirus*	*Siphoviridae*	125114	complete

**94**	Salmonella phage SHP1	KY979109	*Dhakavirus*	*Myoviridae*	62829	complete
**95**	Salmonella phage pSe_SNUABM_01	MN580668	*Tequatrovirus*	*Myoviridae*	172360	complete
**96**	Salmonella virus S16	NC_020416	*Gelderlandvirus*	*Myoviridae*	160221	complete
**97**	Salmonella virus SPN3US	NC_027402	*Seoulvirus*	*Myoviridae*	240413	complete
**98**	Salmonella virus SSE121	NC_027351	*Seunavirus*	*Myoviridae*	147745	complete
**99**	Salmonella phage vB_SenM_SB18	MK759884	*Kolesnikvirus*	*Myoviridae*	85311	complete
**100**	Salmonella virus BPS15Q2	NC_031939	*Felixounavirus*	*Myoviridae*	89817	complete
