# Supplementary figures and images for: Genomic and functional characterization of five novel Salmonella-targeting bacteriophages
Source: Virol J. 2021 Sep 8;18:183. doi: 10.1186/s12985-021-01655-4 (PMC8425127; doi:10.1186/s12985-021-01655-4)

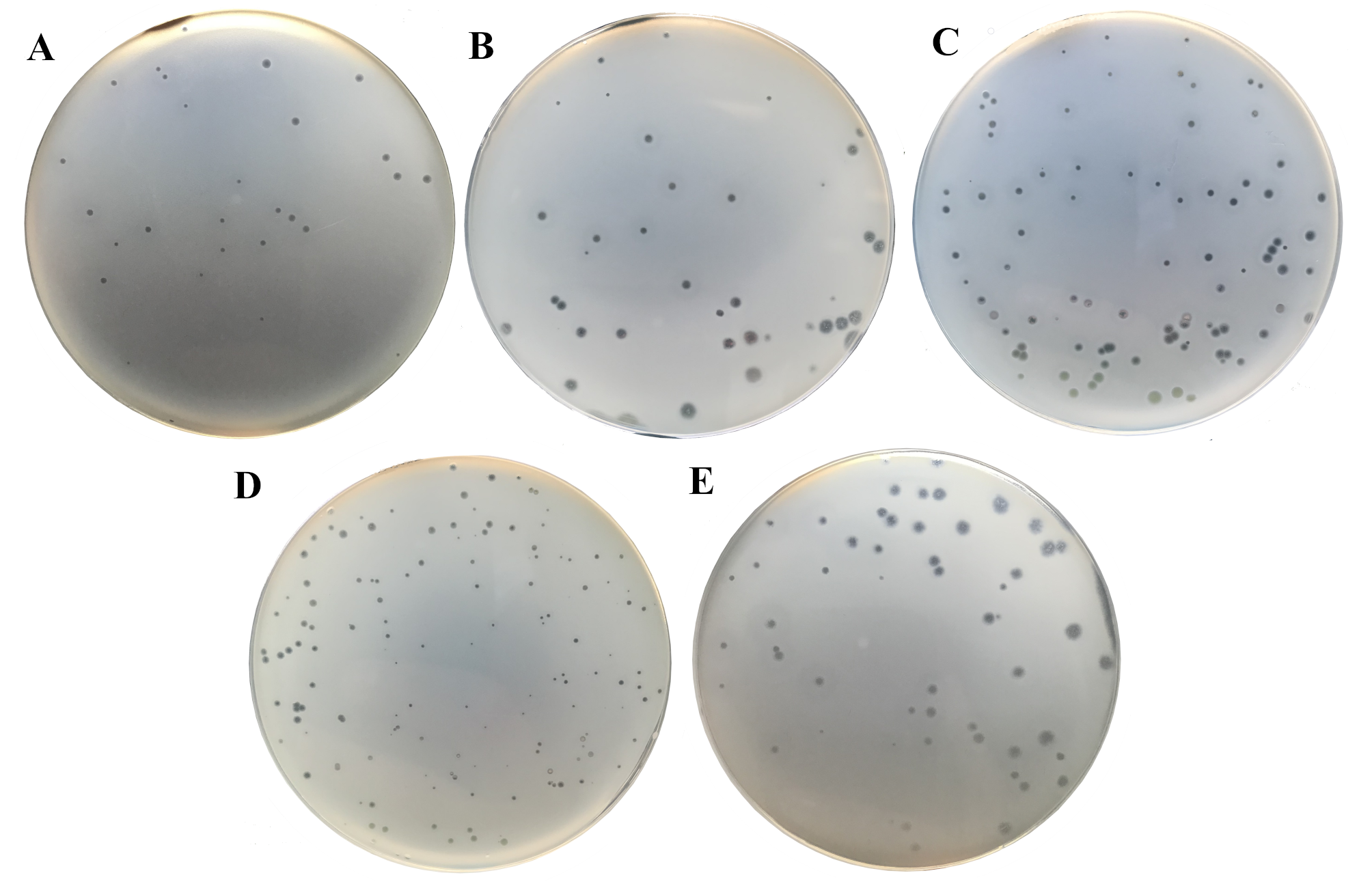

Supplement: Supplementary file 3 — Additional file 3. Fig. S1: UPWr_S1 (a), UPWr_S2 (b), UPWr_S3 (c), UPWr_S4 (d) and UPWr_S5 (e) phages’ plaque morphology on Salmonella Enteritidis lawn. Analysis of plaque morphology revealed that the plaque morphology of each phage was similar, with medium size and a light halo around them. Diameters of plaques were measured manually and diameters of plaques for phages UPWr_S1, UPWr_S2, UPWr_S3, UPWr_S4 and UPWr_S5 were 1.04 mm +/− 0.28 mm, 1.71 mm +/− 0.21 mm, 1.72 mm +/− 0.26 mm, 1.58 mm +/− 0.32 mm, 2.81 mm +/− 0.32 mm, respectively. [file 12985_2021_1655_MOESM3_ESM.tif]

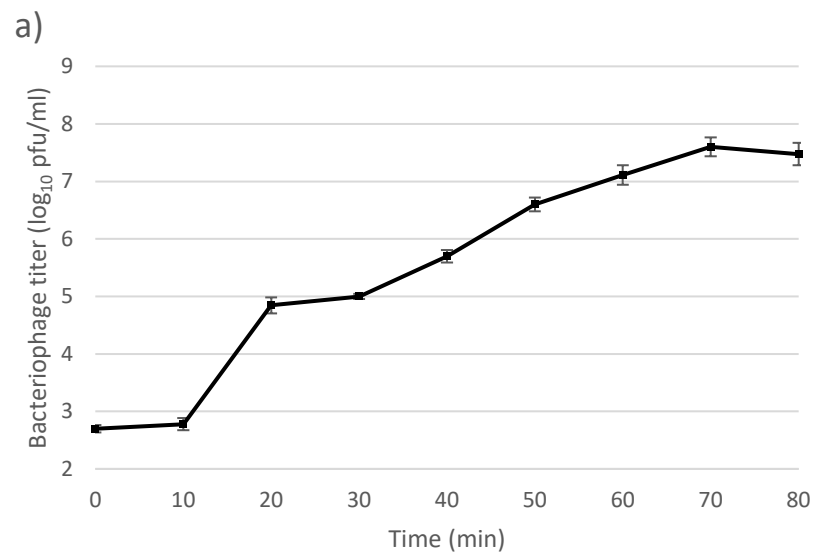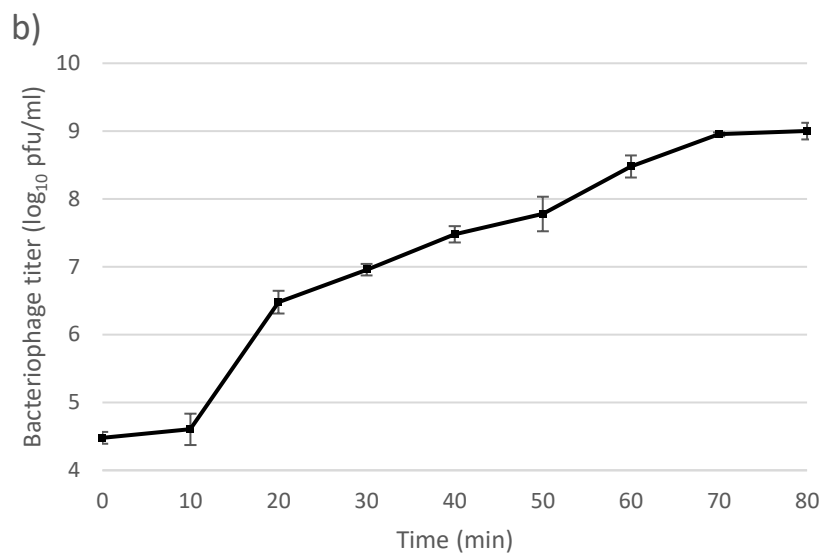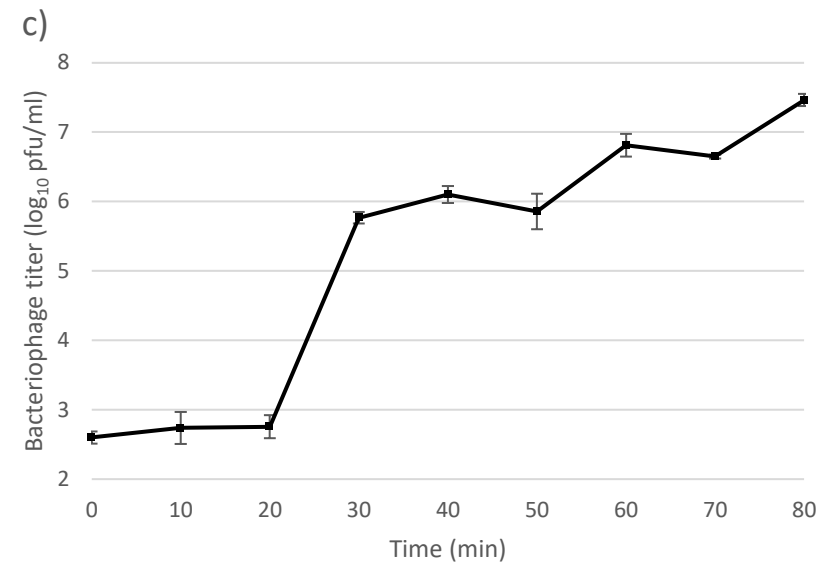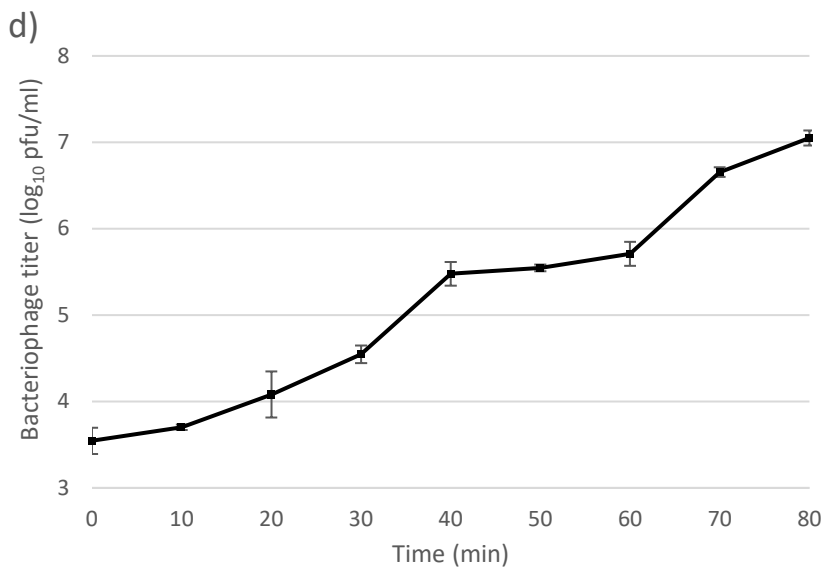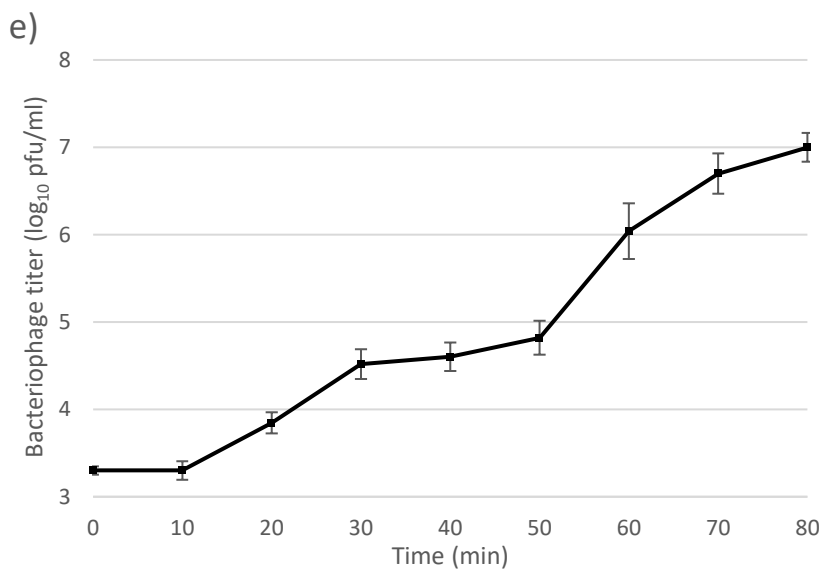

Supplement: Supplementary file 4 — Additional file 4. Fig. S2: One-step growth curves of UPWr_S phages. The one-step growth curve of UPWr_S phages propagated on their respective hosts in LB medium revealed that the latent periods and burst sizes were approximately 15, 12, 9, 24, 23 and 92 minutes for phages (a) UPWr_S1, (b) UPWr_S2, (c) UPWr_S3, (d) UPWr_S4 and (e) UPWr_S5, respectively. The average burst size was estimated to be 201, 89, 92, 48, 92 PFU/cell for phages UPWr_S1, UPWr_S2, UPWr_S3, UPWr_S4 and UPWr_S5, respectively. [file 12985_2021_1655_MOESM4_ESM.pdf]

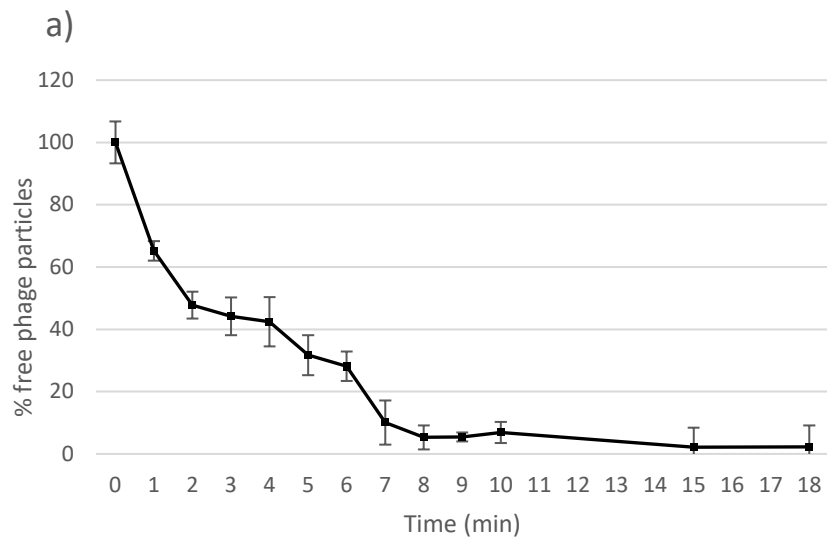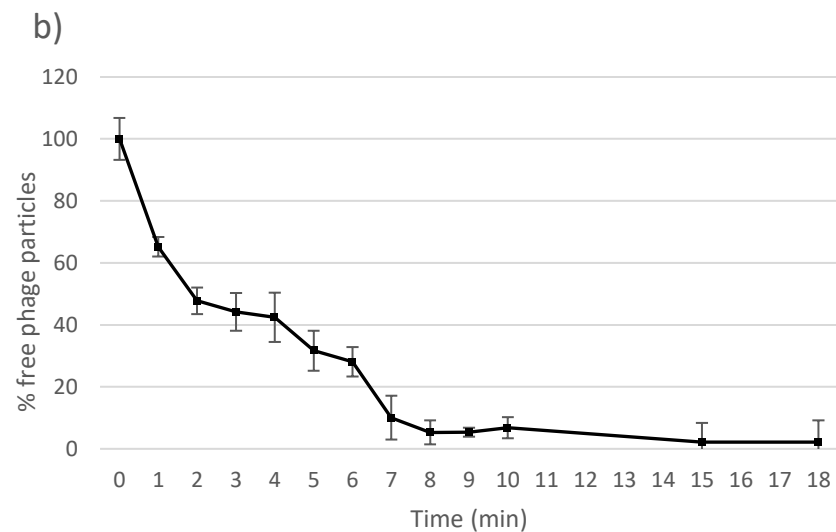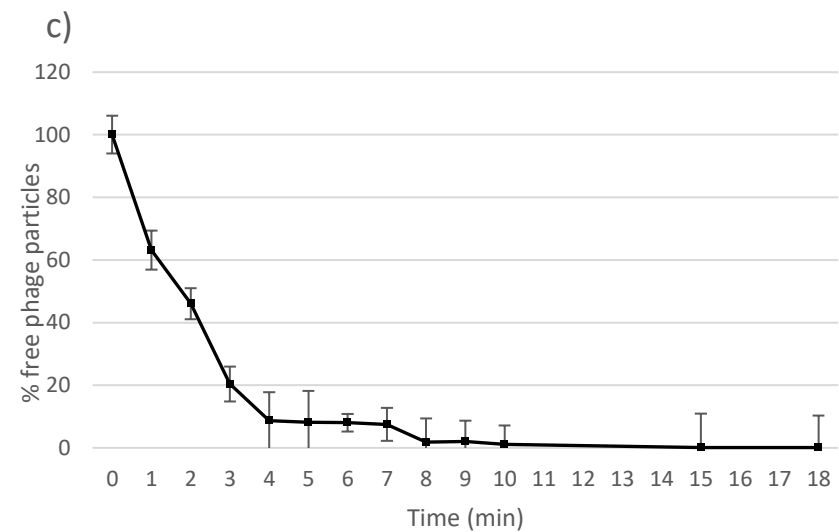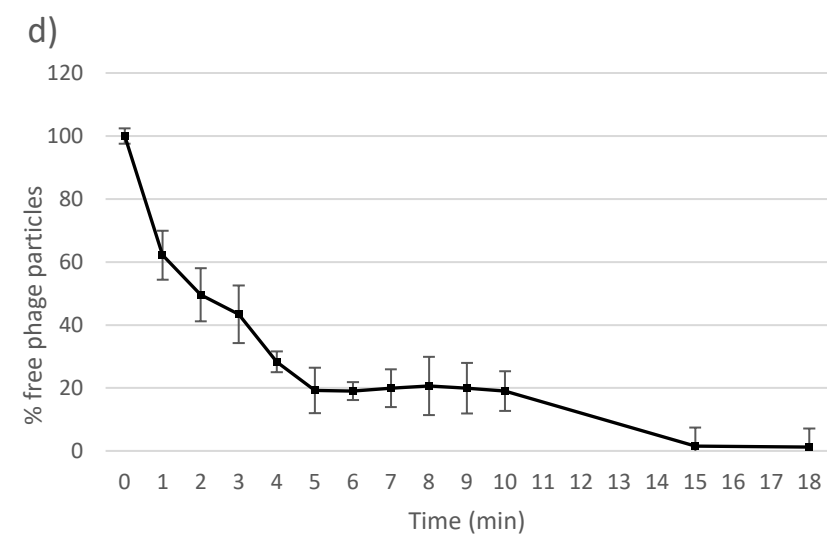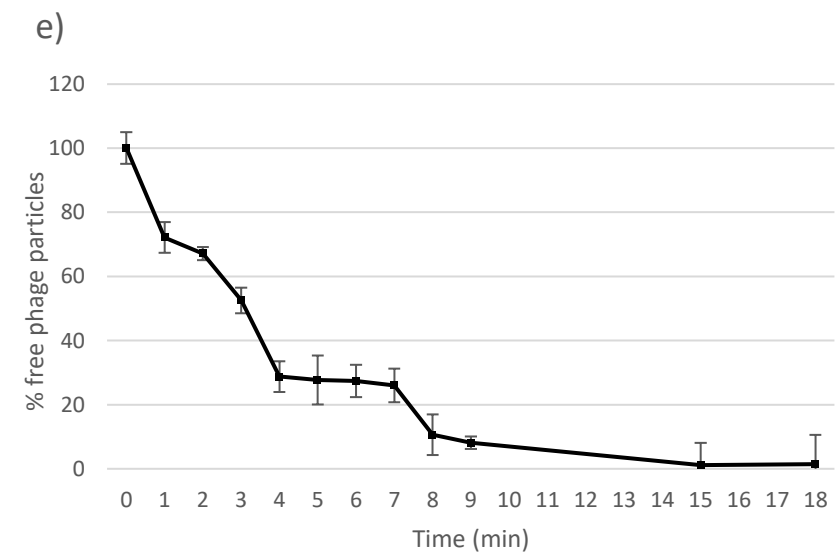

Supplement: Supplementary file 5 — Additional file 5. Fig. S3: Adsorption curves of UPWr_S phages. Adsorption assays showed that the adsorption rates within 10 minutes for phages (a) UPWr_S1, (b) UPWr_S2, (c) UPWr_S3, (d) UPWr_S4 and (e) UPWr_S5 were 90, 93, 99, 80 and 92%, respectively. [file 12985_2021_1655_MOESM5_ESM.pdf]

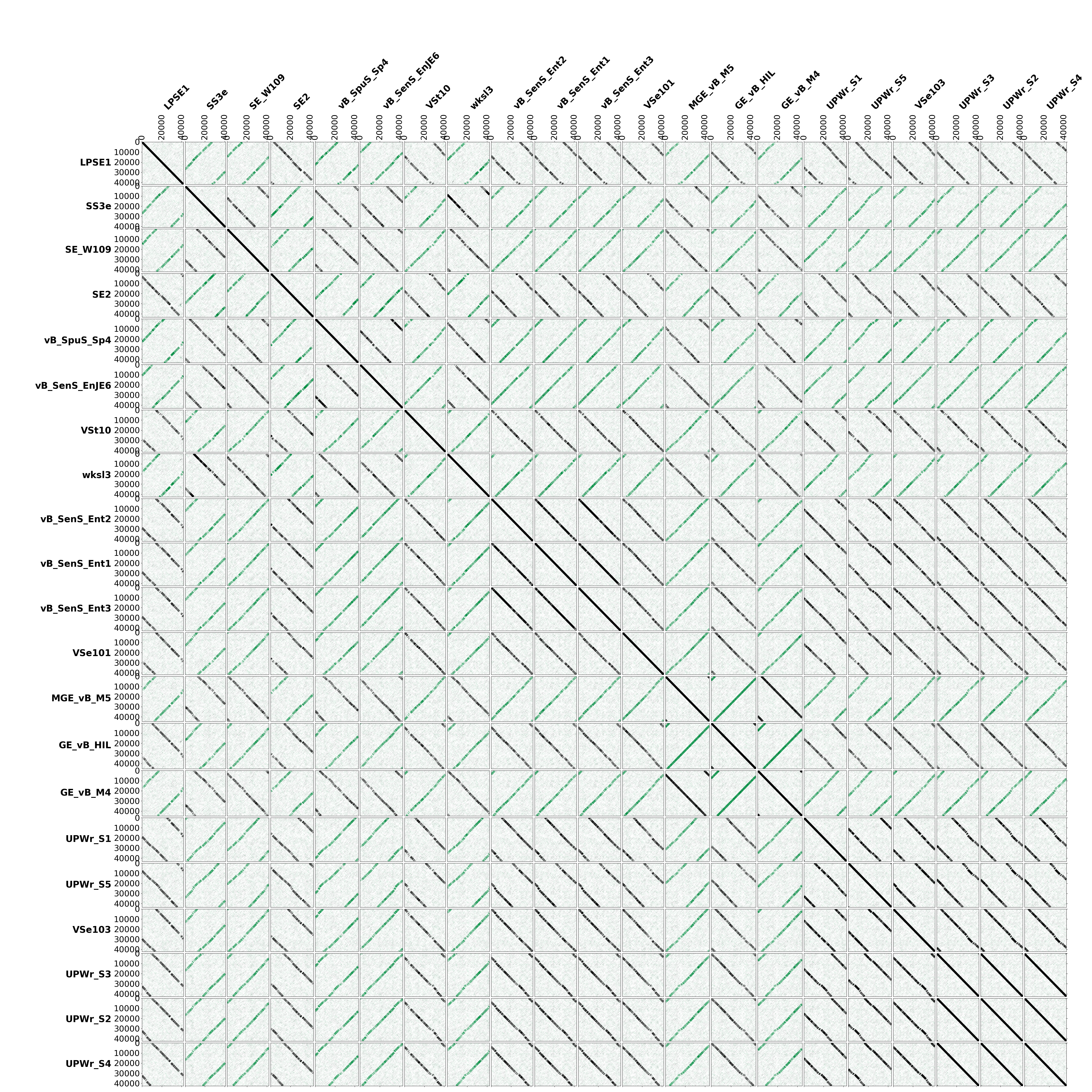

Supplement: Supplementary file 7 — Additional file 7. Fig. S4: Polydot plot comparison of newly sequenced genomes with selected Salmonella phages. All-against-all genome sequence dot plot comparisons of UPWr_S1-5 bacteriophages with selected Salmonella phages belonging to clusters 2 and 3 were performed using FlexiDot. When the DNA residues of both sequences match at the same location on the plot, a dot is drawn at the corresponding position. Once the dots have been plotted, they will combine to form lines that correspond to similar fragments of the genomes. On the main diagonal the sequence’s alignment with itself is presented. [file 12985_2021_1655_MOESM7_ESM.png]
