# Supplementary material for: Genomic and functional characterization of five novel Salmonella-targeting bacteriophages
Source: Virol J. 2021 Sep 8;18:183. doi: 10.1186/s12985-021-01655-4 (PMC8425127; doi:10.1186/s12985-021-01655-4)
Supplement: Supplementary file 6 — Additional file 6. Table S2: Predicted ORFs and genes encoded by the UPWr_S phage genomes. Protein sequences of the predicted ORFs of UPWr_S phages were subjected to the BLASTp program to analyze their best known matches on the NCBI website (https://blast.ncbi.nlm.nih.gov). The average nucleotide identity and query coverage were calculated by BLASTp with the cutoff E-value set at 1E-04. “Related phages” refers to a top hit from NCBI BLASTp. [file 12985_2021_1655_MOESM6_ESM.pdf]

Additional file 6: Table S2. Predicted ORFs and genes encoded by the UPWr_S phage genomes

Gene name	Present in UPWr_S	Length (aa)	Predicted function	Query Cover	Percent Identity	E value	Related phage	GenBank Accession Number
DNA packaging and structural proteins								
gp02	1,2,3,4,5	131	HK97 gp10 family phage protein	100.00%	100.00%	8.00E-92	HK97 gp10	WP_015984941.1
gp03	1,2,3,4,5	139	tail protein	100.00%	99.28 %	2.00E-96	horsemountain	QIO03697.1
gp04	1,2,3,4,5	390	putative tail protein	100.00%	92.24%	0	vB_StyS-sam	LC507823.1
gp10	1,2,3,4,5	138	tail assembly chaperone	100.00%	97.83 %	5.00E-95	vB_SenS_PVP-SE2	AST15484.1
gp12	1,2,3,4,5	777	tail tape measure protein	100.00%	98.84%	0	VSe103	MH424443.1
gp16	1,2,3,4,5	844	tail fiber protein	100.00%	96.45%	0	VSe103	MH424443.1
gp17	1,2,3,4,5	684	tail spike protein	100.00%	96.45%	0	VSe103	MH424443.1
gp45	1,2,3,4,5	489	putative head decoration protein	100.00%	98.04%	0	VSe103	MH424443.1
gp47	1,2,3,4,5	619	intein-containing capsid morphogenesis protein	100.00%	96.61%	0	vB_StyS-sam	LC507823.1

gp48	1,2,3,4,5	152	putative decoration protein	100.00%	98.37%	0	vB_SenS-Ent1	HE775250.1
gp51	1,2,3,4,5	233	scaffold protein	100.00%	99.86%	0	VSe103	MH424443.1
gp52	1,2,3,4,5	348	major capsid protein	100.00%	95.62%	0	vB_SenS_AG11	NC_041991.1
gp53	1,2,3,4,5	74	putative head protein fragment	100.00%	92.44%	2.00E-83	vB_SpuP_Spp11	MN722429.1
gp54	1,2,3,4,5	119	neck whiskers protein fragment	95.00%	91.88%	3.00E-132	SS8	MK972706.1
gp01	1	47	putative head-tail joining protein/ fragment	100.00%	97.92%	1.00E-62	S142	MH370385.1
gp70	1	169	Tail fiber protein fragment	100.00%	96.08%	0	VSe103	MH424443.1
DNA replication and modification								
gp07	1,2,3,4,5	163	HNH homing endonuclease fragment	100.00%	99.59%	0	vB_StyS-sam	LC507823.1
gp08	1,2,3,4,5	384	putative recombination endonuclease subunit protein	100.00%	99.65%	0	SenS-Ent1	HE775250.1
gp21	1,2,3,4,5	821	putative intein containing helicase precursor	100.00%	98.78%	0	SenS-Ent1	HG934470.1
gp24	1,2,3,4,5	1032	DNA polymerase	100.00%	98.10%	0	VSe103	MH424443.1
gp25	1,2,3,4,5	208	DNA polymerase [Salmonella phage BPS11Q3]	100.00%	100.00%	3.00E-152	BPS11Q3	YP_009322844.1
gp30	1,2,3,4,5	728	DNA helicase	100.00%	98.49 %	0	VSe103	AXQ70173.1

gp43	1,2,3,4,5	181	terminase small subunit elongated	100.00%	98.35%	0	VSe103	MH424443.1
gp44	1,2,3,4,5	423	terminase large subunit	100.00%	99.61%	0	VSe103	MH424443.1
gp63	2,3,4	173	homing endonuclease fragment	98.00%	86.68%	1.00E-158	SE-W109	KX649889.1
gp20	1,5	170	DNA primase	100.00%	91.18 %	2.00E-113	vB_EcoS_XY1	QIG59265.1
Transcription and regulation								
gp05	1,2,3,4,5	223	putative DNA-binding protein	100.00%	96.41 %	6.00E-158	vB_SenS-EnJE1	QGJ84396.1
gp29	1,2,3,4,5	72	XRE family transcriptional regulator	100.00%	100.00%	4.00E-109	sidste	MT074481.1
gp32	1,2,3,4,5	56	helix-turn-helix domain-containing protein	100.00%	100.00%	1.00E-33	Shelanagig	QEG07385.1
gp67	5	227	DNA-cytosine methyltransferase	100.00%	89.80%	0	FSL SP-101	NC_042065.1
Host lysis								
gp38	1,2,3,4,5	162	Lysin and holin class II	100.00%	95.91%	0	VSe103	MH424443.1
Superinfection exclusion								
gp09	1,2,3,4,5	59	superinfection immunity protein [*Salmonella* enterica]	100.00%	100.00%	3.00E-32	vB_SpuP_Spp11	WP_015984947.1
Enzymatic function								
gp14	1,2,3,4,5	171	enolase-like protein	100.00%	97.66 %	1.00E-119	FSL SP-101	YP_009617945.1

Unknown function			
gp06	1,2,3,4,5	76	hypothetical protein
gp11	1,2,3,4,5	119	hypothetical protein
gp13	1,2,3,4,5	166	hypothetical protein
gp15	1,2,3,4,5	121	hypothetical protein
gp18	1,2,3,4,5	30	hypothetical protein
gp22	1,2,3,4,5	63	hypothetical protein
gp23	1,2,3,4,5	95	hypothetical protein
gp33	1,2,3,4,5	67	hypothetical protein
gp34	1,2,3,4,5	121	hypothetical protein
gp35	1,2,3,4,5	144	hypothetical protein
gp36	1,2,3,4,5	108	hypothetical protein
gp37	1,2,3,4,5	96	hypothetical protein
gp39	1,2,3,4,5	61	hypothetical protein
gp40	1,2,3,4,5	51	hypothetical protein
gp41	1,2,3,4,5	118	hypothetical protein
gp42	1,2,3,4,5	99	hypothetical protein
gp46	1,2,3,4,5	216	hypothetical protein
gp49	1,2,3,4,5	128	hypothetical protein
gp50	1,2,3,4,5	47	hypothetical protein

gp55	1,2,3,4,5	62	hypothetical protein
gp56	1,2,3,4,5	169	hypothetical protein
gp57	1,2,3,4,5	201	hypothetical protein
gp26	1,2,3,4	58	hypothetical protein
gp59	2,3,4,5	62	hypothetical protein
gp60	2,3,4,5	119	hypothetical protein
gp61	2,3,4	51	hypothetical protein
gp62	2,3,4	477	hypothetical protein
gp64	2,3,4	90	hypothetical protein
gp65	2,3,4	176	hypothetical protein
gp66	2,3,4	77	hypothetical protein
gp19	1,5	51	hypothetical protein
gp28	1,5	149	hypothetical protein
gp31	1,5	77	hypothetical protein
gp27	1	475	hypothetical protein
gp58	1	119	hypothetical protein
gp68	5	414	hypothetical protein
gp69	5	217	hypothetical protein
